# Comprehensive Comparison of Surgery Followed by Radiotherapy and Radical Radiotherapy for Cervical Cancer: A Multicenter Retrospective Propensity-Score-Matched Analysis

**DOI:** 10.3390/cancers18050865

**Published:** 2026-03-07

**Authors:** Junyi Liu, Youwen Zhu, Kun Liu, Dongfeng Deng, Qiuping Yang, Weisong Wang, Xianyu Liu, Hong Zhu

**Affiliations:** 1 Department of Radiation Oncology, Xiangya Hospital, Central South University, Changsha 410008, China; 2 National Clinical Research Center for Geriatric Disorders, Xiangya Hospital, Central South University, Changsha 410008, China; 3 Department of Oncology, Hunan University of Medicine General Hospital, Huaihua 418000, China; 4 Department of Pathology, Tangshan Cancer Hospital, Tangshan 063300, China; 5 Department of Cardiology, The First Hospital of Hunan University of Chinese Medicine, Changsha 410007, China; 6 Department of Oncology, Zhuzhou 331 Hospital, Zhuzhou 412002, China

**Keywords:** cervical cancer, surgery, radiotherapy, efficacy, cost-effectiveness

## Abstract

**Background:** While surgery and radiotherapy are the standard of care for patients with cervical cancer (CC), debate persists regarding the choice of whether treatment should consist of surgery followed by radiotherapy or initial direct radical radiotherapy. The present study was therefore devised to compare real-world clinical outcomes and economic assessments associated with these different treatment approaches. **Methods:** Six tertiary medical centers retrospectively identified patients with International Federation of Gynecology and Obstetrics (FIGO) 2018 stage I-IVA CC who underwent surgery followed by radiotherapy (surgery–radiotherapy group) or radical radiotherapy (radiotherapy group) between 2015 and 2023 in China. The progression-free and overall survival (PFS and OS) of these patients were compared using Kaplan–Meier and propensity-score-weighted proportional risk models. Economic analyses were also conducted based on patient follow-up for up to 8 years from the start of treatment. **Results:** A total of 980 patients receiving surgery–radiotherapy and radiotherapy were identified for matching. Propensity score weighting revealed no significant statistical differences in PFS (hazard ratio [HR], 0.75; 95% confidence interval [CI], 0.44–1.28; *p* = 0.29) and OS (HR, 0.49; 95% CI, 0.20–1.21; *p* = 0.12) when comparing these groups. Subgroup analysis found differences in PFS (HR, 0.17; 95% CI, 0.04–0.77; *p* = 0.02) among adenocarcinoma. Economic analyses revealed that the incremental cost-effectiveness ratio of the surgery–radiotherapy group versus the radiotherapy group was $40,831/quality-adjusted life-year (QALY), which is higher than the Chinese willingness-to-pay threshold of $35,841/QALY. **Conclusions:** Survival outcomes were similar for patients with CC who underwent surgery–radiotherapy and radiotherapy. Further, radical radiotherapy may be cost-effective for such patients considering economic factors in China.

## Simple Summary

Medical guidelines generally recommend surgery for early-stage cervical cancer and radiation therapy for advanced stages; however, personal preferences and other factors often lead some patients to choose surgery followed by radiation instead of starting directly with radiation therapy. This study aimed to compare the medical success, patient safety, and financial costs of these two treatment paths using real-world patient information. The results showed that patients experienced similar survival rates regardless of which of the two treatments they received. However, starting directly with comprehensive radiation therapy proved to be a better financial value for patients in China. By demonstrating that both methods are equally effective for survival but differ in overall cost, this research provides valuable information to help doctors personalize care for their patients. Furthermore, it offers clear data to assist national health insurance programs in making better coverage decisions, ensuring that medical resources are used wisely and efficiently to benefit society as a whole.

## 1. Introduction

Cervical cancer is the fourth most common and third deadliest cancer in women, with approximately 662,301 new diagnoses and 348,874 deaths globally in 2022, with approximately 60% of these cases occurring in Asia, including 23% in China [1]. Early cervical cancer is primarily treated via radical total hysterectomy with or without pelvic lymph node dissection. While the prognosis of these patients tends to be excellent, with a 5-year overall survival (OS) rate exceeding 92%, certain postoperative risk factors including tumor size, paracentral infiltration, lymph node metastasis, and lymphatic vascular space infiltration (LVSI) have been linked to greater odds of tumor recurrence [2,3,4,5,6]. Postoperative adjuvant radiotherapy with or without chemotherapy is generally employed as an approach to treat these patients, linked to a reduction in local recurrence with no significant improvements in patient survival outcomes [6,7].

External beam radiotherapy (EBRT) with brachytherapy alone or together with chemotherapy has been demonstrated in randomized trials to be similar to surgery in terms of efficacy for inoperable patients [8]. This treatment strategy is also suggested for individuals with locally advanced disease [6]. While the approval of surgery followed by radiotherapy with or without chemotherapy (surgery–radiotherapy) and radiotherapy with or without chemotherapy (radiotherapy) can be effective approaches to cervical cancer management, significant differences in terms of available treatment options are evident for patients with different types of cervical cancer in clinical settings owing to the patient’s preference for treatment and the lack of detailed examination results before treatment. Whether postoperative radiotherapy or radical radiotherapy is more appropriate for the treatment of cervical cancer remains a matter of controversy. There have not been any prospective ‘head-to-head’ studies to date directly comparing the outcomes associated with surgery–radiotherapy and radiotherapy treatment strategies across all patients with cervical cancer.

Owing to this persistent uncertainty regarding the relative clinical benefits of these different interventional strategies, the present study was developed to leverage real-world population data as a means of comparing the clinical efficacy and safety of surgery–radiotherapy versus radiotherapy as approaches to cervical cancer management in China. Given the rising costs associated with cancer treatment, there is also increasing interest from healthcare decision-makers and health technology agencies in life-cycle health technology assessments as a means of comparing the relative cost-effectiveness of different treatment strategies, thereby providing a foundation for informed decision-making regarding clinical management and treatment strategies.

## 2. Materials and Methods

### 2.1. Data Source and Study Population

The clinical database of patients newly diagnosed with International Federation of Gynecology and Obstetrics (FIGO) 2018 stage I-IVA cervical cancer who underwent surgery–radiotherapy or radiotherapy treatment between 2015 and 2023 was reviewed based on the inpatient and outpatient records of the Department of Oncology or Gynecology from six grade A tertiary medical centers, with the primary site of these analyses being Xiangya Hospital of Central South University (Supplementary Table S1). This study included patients with (1) histologically confirmed cervical cancer, (2) detailed clinical data, and (3) treatment based on radiotherapy or surgery followed by radiotherapy. Patients were excluded if they underwent incomplete radiotherapy, incomplete hysterectomy, or immunotherapy (Supplementary Figure S1), but patients who died during the study or were lost to follow-up were eligible for inclusion. Clinical information was independently collected by two researchers, with final approval from a third senior investigator. The Institutional Review Committee of Xiangya Hospital of Central South University approved this study (ethics approval number: 202402038), which waived the need for informed patient consent.

### 2.2. Study Design and Clinical Assessments

This was a retrospective cohort study conducted across 6 sites in China. Patients included in this study underwent modified radical hysterectomy or radical hysterectomy with or without lymph node dissection followed by intensive modulated radiation therapy (IMRT, 45 Gy in 25 fractions) with or without chemotherapy (cisplatin [40 mg/m^2^], carboplatin [area under the concentration-time curve, 2 mg per milliliter per minute], or nimotuzumab [200 mg]) every week limited to 6 cycles followed by intracavitary/interstitial brachytherapy (three-dimensional high-dose-rate brachytherapy, 28 Gy in 4 fractions) with or without neoadjuvant or adjuvant chemotherapy (paclitaxel [175 mg/m^2^ of body-surface area] and either cisplatin [75 mg/m^2^] or carboplatin [area under the concentration-time curve, 5 mg per milliliter per minute] at the oncologist’s discretion, limited to 4 cycles) every 3 weeks as per our practice guidelines (surgery–radiotherapy group) or underwent this same treatment without surgery (radiotherapy group). After treatment was complete, these patients were routinely monitored every 3 months for 3 years and once annually thereafter. MRI or CT scans were conducted when there was clinical concern regarding potential metastasis or disease recurrence. Tumor recurrence was detected based on the RECIST 1.1 criteria by imaging or biopsy findings. Primary study outcomes were OS, measured as the interval between surgery or radiotherapy initiation and all-cause death, and progression-free survival (PFS), measured as the interval between surgery or radiotherapy initiation and disease recurrence, metastasis, or death. Survival was measured from the day when the treatment began until death or the most recent follow-up.

Both radiation-induced and non-radiation-induced adverse events were classified as per the Common Terminology Criteria for Adverse Events (CTCAE version 5.0) and Radiation Therapy Oncology Group/European Organisation for Research and Treatment of Cancer (RTOG/EORTC). Events not related to radiation were recorded when patients were undergoing trial treatments, while adverse events related to radiation, which primarily included cystitis and enteritis, were classified as either acute or late toxicities. Acute toxicity was assessed during radiotherapy and within 3 months after radiotherapy. Late toxicity was defined as adverse events occurring after 90 days from completion of radiotherapy.

### 2.3. Statistical Analysis

Categorical and continuous variables were compared between the surgery–radiotherapy and radiotherapy groups with chi-square tests and Wilcoxon tests, respectively, reporting the corresponding data as medians (range) and numbers (%). Given the retrospective nature of these analyses and their inevitable susceptibility to selection bias, a propensity-score-weighted proportional risk model was employed to reduce any potential confounding. The utilized propensity scores were based on a logistic regression predictive model for the surgery–radiotherapy group using potential confounding factors including age, stage, pathological type, differentiation grade, lymph node status, human papillomavirus (HPV) status, and combination therapy. Propensity score matching was performed at a 3:1 ratio between the radiotherapy and surgery–radiotherapy groups, employing a nearest-neighbor matching strategy and a caliper width of 0.2 using R (v 4.3.1) with the ‘MatchIt’ package [9]. Differences in the adjusted covariates were then analyzed between groups, with *p*-values greater than or equal to 0.05 generally being considered indicative of acceptable balance [10]. Kaplan–Meier curves and stratified log-rank tests were used to compare OS and PFS outcomes between groups in this propensity-score-matched population, with further exploratory subgroup analyses. HRs and 95% CIs were analyzed with a stratified Cox proportional hazards model. Likelihood ratio tests were used to calculate *p*-values, with *p* < 0.05 being regarded as significant.

### 2.4. Cost-Effectiveness Analysis

The Consolidated Health Economic Evaluation Reporting Standards (CHEERS 2022) statement was used to guide the economic analyses conducted herein (Supplementary Table S3) [11]. Total costs from the index data until death or the end of an 8-year follow-up interval were calculated from the payers’ perspective. Total per-patient costs were calculated by summing together costs associated with treatment, hospitalization, administrative costs, imaging tests, laboratory tests, and adverse event management. Prices were reported in US dollars based on an exchange rate of $1 = ¥7.1984 (February 2024). Costs were derived from the average cost data from tertiary care centers (Supplementary Table S5). The utility was used to reflect patients’ quality-of-life (QoL) weights in the natural history of the disease, on a scale of 0 (death) to 1 (full health). Utilities were used to obtain QALYs by discount Lys [12]. Life-years (LYs) and quality-adjusted LYs (QALYs) served as the primary indicators of therapeutic efficacy. LYs were defined based on 8-year survival data from the index data until death or the end of this follow-up period, whereas QALYs were measured by adjusting the 8-year survival rates of patients from these two treatment groups for PFS and progressive disease (PD). Utility weights were derived from literature sources and included a time-weighted utility of 0.82 for patients receiving initial treatment and 0.65 for patients experiencing PD [13,14] (Supplementary Table S5).

As the passage of time influences both cost and efficacy considerations, a 5% discounting rate was applied for these analyses [15]. The incremental cost-effectiveness ratio (ICER) was used as a measure of cost-effectiveness and was calculated by dividing the incremental cost when comparing the two treatment groups by the difference in overall efficacy between these groups (LYs or QALYs gained) [16]. This ICER was then compared to the Chinese willingness-to-pay (WTP) threshold of $35,841/QALY, which is 3 times the Chinese GDP per capita [17]. The odds of a treatment being cost-effective were assessed with 10,000 Monte Carlo simulations and through the plotting of acceptability curves and scatter plots [18]. Treatment cost-effectiveness was also assessed for major patient subgroups.

## 3. Results

### 3.1. Patients and Treatment

From 16 March 2015 to 29 May 2023, a total of 991 patients were identified, 11 of whom were ultimately excluded from this study (Supplementary Figure S1). The remaining 980 patients were separated into a radiotherapy group (780 patients) and a surgery–radiotherapy group (200 patients) based on the treatments they underwent. Propensity score matching led to the identification of 380 and 140 patients in the radiotherapy and surgery–radiotherapy groups, respectively, who were retained for analysis. The resultant matched patient population exhibited baseline demographic and clinical characteristics that were largely balanced across groups, with an overall mean age of 53 (range, 24–82) years, 88.9% incidence of squamous-cell carcinoma, and 63.7% incidence of HPV positivity. The mean dose of EBRT to the pelvis with or without the abdomen, EBRT to lymph node metastases, and brachytherapy to the primary lesion (EQD2) were 45 Gy, 60 Gy, and 34 Gy, respectively (Table 1 and Table S2).

The median follow-up interval, defined as the period from initial treatment to the data cutoff date (10 January 2024), was 46.2 months (IQR, 21.4–72.1), with the longest follow-up interval being approximately 8.75 years. As of the final analysis, 30 patients (5.8%) in the surgery–radiotherapy and radiotherapy groups had died, while 490 patients were censored without evidence of death (94.2%; 391 [75.2%] alive, 99 [19.0%] lost to follow-up).

### 3.2. Efficacy

**Figure 1:**
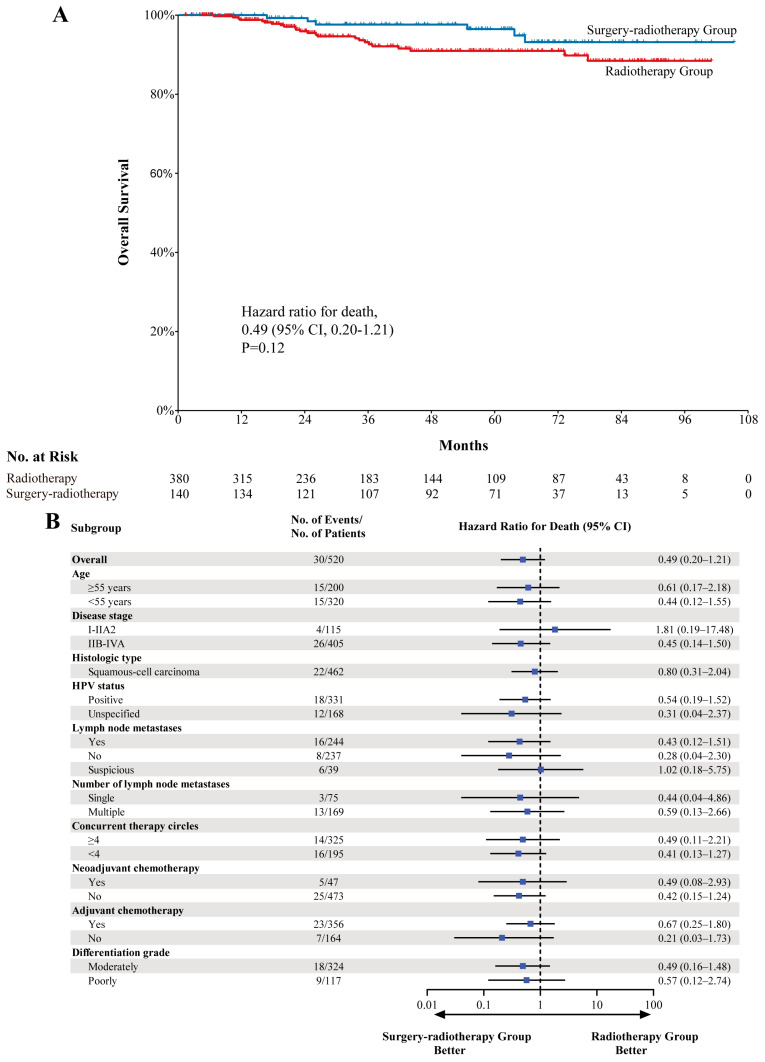
Kaplan–Meier estimates of overall survival. (**A**) overall population. (**B**) subgroup population. Abbreviations: CI, confidence interval; HPV, human papillomavirus.

In the propensity-score-matched population, the median OS endpoint was not reached, and no differences in survival were observed between these groups when controlling for confounding factors (HR, 0.49; 95% CI, 0.20–1.21; *p* = 0.12) (Figure 1). The estimated 5-year OS in the surgery–radiotherapy group was 96.4% (95% CI, 92.9–99.9%), while in the radiotherapy group, it was 90.9% (95% CI, 87.2–92.8%). The estimated 8-year OS in the surgery–radiotherapy group was 93.1% (95% CI, 87.4–98.8%), while in the radiotherapy group, it was 88.4% (95% CI, 83.5–93.3%) (Figure 1). As the HRs were less than 1, these OS analyses tended to favor surgery–radiotherapy treatment in pre-specified patient subgroups, except for patients with FIGO 2018 stage I-IIA2 disease (HR, 1.81; 95% CI, 0.19–17.48; *p* = 0.61) and suspected lymph node metastases (HR, 1.02; 95% CI, 0.18–5.75; *p* = 0.98) (Figure 1 and Supplementary Figure S2).

**Figure 2:**
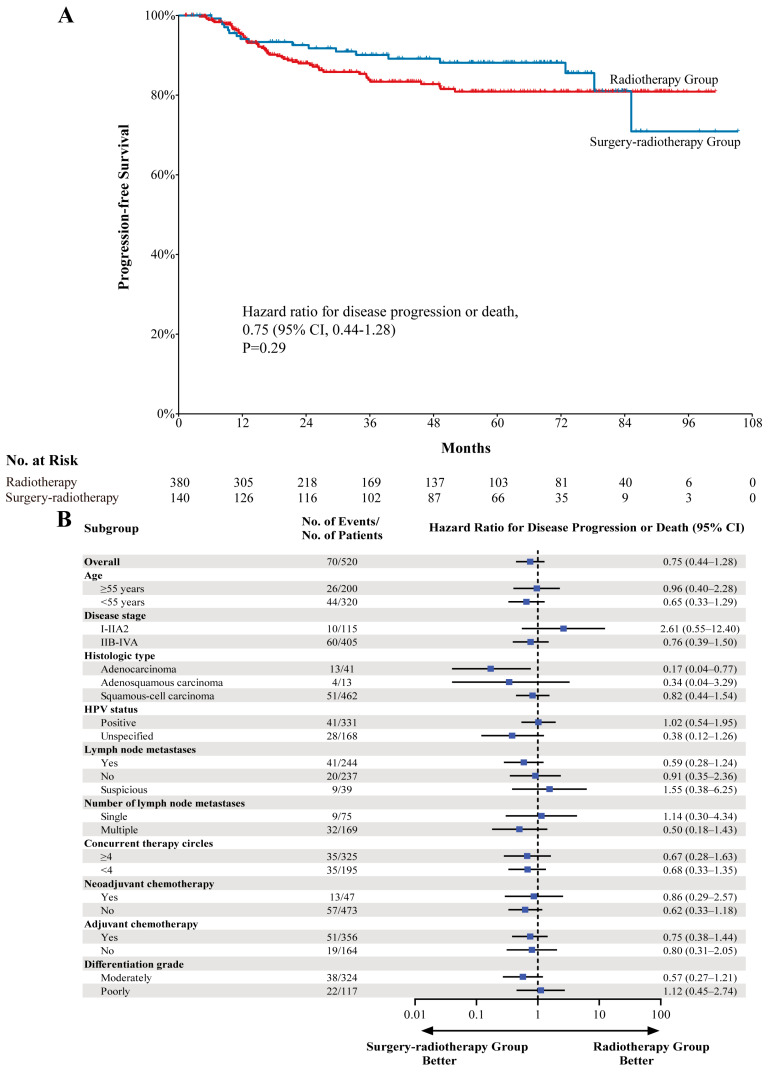
Kaplan–Meier estimates of progression-free survival. (**A**) overall population. (**B**) subgroup population. Abbreviations: CI, confidence interval; HPV, human papillomavirus.

In total, 18 of the patients in the surgery–radiotherapy group (12.9%) died or exhibited disease progression, compared to 45 (11.8%) in the radiotherapy group. These patients did not reach the median PFS endpoint, with no significant difference in PFS between these groups (HR, 0.75; 95% CI, 0.44–1.28; *p* = 0.29) (Figure 2). The respective estimated 5- and 8-year PFS rates in the surgery–radiotherapy group were 88.1% (95% CI, 82.4–93.8%) and 70.9% (95% CI, 49.9–91.9%), compared to 80.9% (95% CI, 76.0–85.8%) and 80.9% (95% CI, 76.0–85.8%) in the radiotherapy group (Figure 2). The respective objective response rate (ORR) and disease control rate (DCR) values were 87.9% (95% CI, 82.4–93.3%) and 87.9% (95% CI, 82.4–93.3%) in the surgery–radiotherapy group, compared to 87.1% (95% CI, 83.7–90.5%) and 88.2% (95% CI, 84.9–91.5%) in the radiotherapy group (Supplementary Table S4). The HR for PFS did not exceed 1 in the majority of the specified patient subgroups, other than patients with FIGO 2018 stage I-IIA2 disease (HR, 2.61; 95% CI, 0.55–12.40; *p* = 0.23), suspected lymph node metastases (HR, 1.55; 95% CI, 0.38–6.25; *p* = 0.54), single lymph node metastasis (HR, 1.14; 95% CI, 0.30–4.34; *p* = 0.85), and poorly differentiated disease (HR, 1.12; 95% CI, 0.45–2.74; *p* = 0.81) (Figure 2 and Supplementary Figure S3).

### 3.3. Adverse Events

The median durations of treatment for patients in the surgery–radiotherapy and radiotherapy groups were 4.6 months (range, 2.5–6.0 months) and 4.0 months (range, 1.5–5.8 months), respectively. Adverse events of any cause affected 98.3% of the enrolled patients in the surgery–radiotherapy group (50.0% for grade 3 or higher) and 99.4% of patients in the radiotherapy group (74.1% for grade 3 or higher) (Table 2). The most frequently encountered non-radiation-induced adverse events were anemia, leukopenia, hypoalbuminemia, neutropenia, and thrombocytopenia in both groups. The most frequently encountered grade 3 or higher non-radiation-induced adverse events were leukopenia (38.1% in the surgery–radiotherapy group vs. 51.0% in the radiotherapy group), neutropenia (28.8% vs. 33.1%), anemia (11.0% vs. 20.1%), and thrombocytopenia (1.7% vs. 9.4%). The most common any grade and grade 3+ higher radiation-induced adverse events were acute enteritis (28.0% vs. 45.7% for any grade and 5.1% vs. 11.6% for grade 3 or higher), late enteritis (11.0% vs. 30.9% and 1.7% vs. 1.9%), late cystitis (6.8% vs. 27.0% and 1.7% vs. 13.5%), and acute cystitis (32.2% vs. 21.2% and 0.9% vs. 0.6%). No adverse-event-related deaths were observed in either group.

### 3.4. Cost-Effectiveness Result

**Figure 3:**
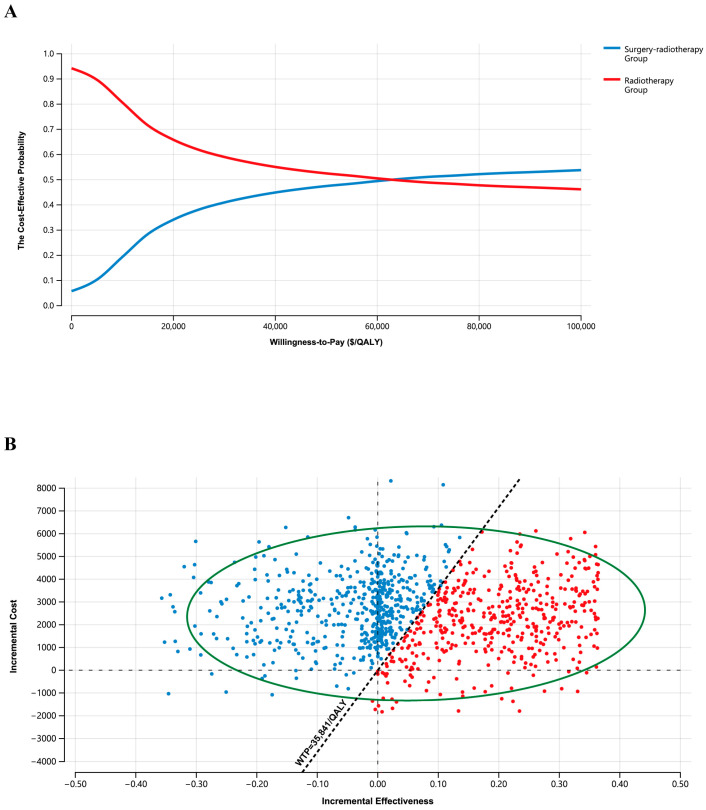
The probability of cost-effectiveness. (**A**) Cost-effectiveness acceptability curves. When the two curves intersect, the probability of cost-effectiveness for both groups is 50%. The WTP value in China is on the left side of the intersection point; thus, the radiotherapy group is more cost-effective. (**B**). Cost-effectiveness scatter plot. This indicated that the respective odds of the surgery–radiotherapy group and the radiotherapy group being cost-effective at the Chinese WTP threshold of $35,841/QALY were 43.44% and 55.56%, respectively. Abbreviations: WTP, willingness-to-pay; QALY, quality-adjusted life-year.

Patients in the radiotherapy group attained 0.066 incremental LYs (0.8 months) when compared to patients in the surgery–radiotherapy group. When adjusting for 8-year LYs based on utility weights and discounting rates, both the efficacy and costs for patients in the surgery–radiotherapy group (5.396 QALYs and $29,193) were elevated compared to those for patients in the radiotherapy group (5.334 QALYs and $26,667), yielding an ICER of $40,758/QALY (Table 3). Cost-effectiveness acceptability curves and scatter plots indicated that the respective odds of the surgery–radiotherapy group and radiotherapy group being cost-effective at the Chinese WTP threshold of $35,841/QALY were 43.44% and 55.56%, respectively (Figure 3). The radiotherapy treatment strategy was further found to be more cost-effective in the majority of patient subgroups, particularly for those patients with suspected lymph node metastasis, single lymph node metastasis, poorly differentiated disease, and FIGO 2018 stage I-IIA2 disease (Supplementary Table S5).

## 4. Discussion

### 4.1. Results in the Context of What Is Known

Although randomized controlled trials focused on the surgical, radiotherapeutic, and chemotherapeutic management of patients with cervical cancer have been published, controversy remains regarding the optimal treatment strategy for affected patients [7,8,20,21,22,23,24,25]. Selectors of surgical/radiotherapy-based or radiotherapy-based treatments presented their views for the overall population with cervical cancer, but clear evidence regarding which of these approaches is superior in terms of clinical efficacy is still lacking. To overcome this issue, propensity-score-matched data were herein used to analyze the clinical outcomes and economic feasibility of a surgery–radiotherapy group versus a radiotherapy group based on data from a real-world Chinese cervical cancer population. These analyses ultimately revealed that patients in the surgery–radiotherapy group experience similar tumor treatment outcomes to patients in the radiotherapy group, with the latter of these treatments being more cost-effective. The difficulties associated with conducting a true head-to-head prospective trial comparing these two treatment regimens underscores the importance of the results of this real-world multicenter retrospective cohort analysis.

### 4.2. Principal Findings and Clinical Implications

The results of the present retrospective analyses suggest that 5-year (8-year) OS of the surgery–radiotherapy group versus the radiotherapy group was 96.4% (93.1%) versus 90.9% (88.4%) (HR = 0.49). In addition, 5-year (8-year) PFS of the surgery–radiotherapy group versus the radiotherapy group was 88.1% (70.9%) versus 80.9% (80.9%) (HR = 0.75). However, there were no significant differences between these groups. Both the ORR and DCR appeared similar in these two treatment groups, supporting the potential benefits of these treatments to patients with cervical cancer. These results align well with findings from prior trials, revealing no significant differences in the efficacy of radiotherapy alone or in combination with surgery (56–89% vs. 79–80%) when used to treat invasive cervical cancer [20]. Analyses of safety outcomes revealed higher adverse event incidence in the radiotherapy group relative to the surgery–radiotherapy groups without any instances of severe or fatal adverse events. A careful analysis of these data indicates that the adverse reactions caused by radiation in the two groups of patients were more common in the radiotherapy group. This is because the patients in this group still had clearly visible imaging lesions during the radiotherapy planning process, resulting in the organs at risk (such as the rectum and bone marrow) being closer to the tumor target area. Consequently, the radiation dose to these organs was higher, leading to more severe toxic side effects. Moreover, there was a significant difference in the post-treatment dose between the two groups, which led to the adverse reactions caused by radiation being more common in the radiotherapy group. Close attention is thus warranted when assessing differences in the toxicity of particular therapeutic regimens, and there is a need for efforts to continuously improve combination treatment strategies for patients undergoing surgical treatment.

The results of the present economic evaluation suggest that any clinical benefits linked to the surgery–radiotherapy treatment strategy are not cost-effective from the perspective of Chinese payers at the established WTP threshold (ICER, $40,758/QALY). These analyses revealed that patients in the surgery–radiotherapy group exhibited an average 8-year total costs that was 9% above that of patients in the radiotherapy group. The major contributing factors to these costs in both groups were the costs associated with cancer treatment (radiation, surgery, chemotherapy, and nimotuzumab) and the follow-up costs for patients. This is the first cost-effectiveness analysis of this kind to our knowledge, and there have been only a limited number of similar studies conducted to date indicating that radical radiotherapy is a cost-effective approach to the treatment of stage IB2 and IIB cervical cancer when compared to radical surgery alone [26,27]. In real-world settings, a 10% reduction in the cost of radical surgery would place the ICER for the surgery–radiotherapy group below the Chinese WTP threshold of $35,841/QALY. These results provide an evidence base that can be leveraged to aid patient selection, clinical decision-making, and healthcare pricing. The results of this study indirectly highlight the importance of a multidisciplinary team. A comprehensive assessment involving gynecologists, oncology radiation therapists, and oncology physicians is necessary to obtain the most suitable treatment plan for the patient.

### 4.3. Research Implications

Precision cancer treatment has long been an area of intensive interest for both oncologists and researchers. This interest has fueled efforts to identify specific biomarkers and features that can predict patient treatment outcomes, thereby allowing for individualized treatment planning. Prognostic risk factors that have previously been linked to outcomes for patients with cervical cancer include age, tumor size, miscarriage, disease stage, the number of lymph node metastases, and histopathologic parameters [28,29,30,31,32]. However, most comparative analyses of radiotherapy and surgery outcomes have failed to detect any differences in survival for these subgroups [8,20]. In the present study, survival analyses similarly revealed no differences in survival outcomes in most patient subgroups, although significantly better PFS was observed in the surgery–radiotherapy group when specifically focusing on adenocarcinoma patients (HR, 0.17; 95% CI, 0.04–0.77; *p* = 0.02). Furthermore, slightly greater odds of cost-effectiveness were observed for the surgery–radiotherapy group when focusing on adenocarcinoma or adenosquamous carcinoma patients with survival. Recent studies have shown that patients with AC who undergo hysterectomy but do not receive further treatment have a 44% risk of recurrence. In contrast, the recurrence risk is significantly reduced to 9% for patients who receive adjuvant radiotherapy [33]. Therefore, for patients with lymph node metastasis, radiotherapy should be recommended. Given the similar efficacy outcomes between the surgery–radiotherapy group and the radiotherapy group in this study, for locally advanced cervical cancer, new multi-modal treatment concepts still need to be explored. Recent several clinical studies have found that neoadjuvant immunotherapy combined with surgery, concurrent chemoradiotherapy combined with immunotherapy, and concurrent chemoradiotherapy combined with targeted therapy can all improve the prognosis of patients [34,35,36]. Meanwhile, due to the lack of any clinical parameters or biomarkers that can predict the treatment outcome of patients, it will be necessary in the future to conduct a more comprehensive exploration of these clinical features or biomarkers in order to help determine which patients are most likely to benefit from a specific treatment regimen. In addition, the importance of the economic benefits associated with specific treatment options deserves to be stressed.

### 4.4. Strengths and Limitations

This study is the first to our knowledge to have leveraged population-based patient-level data to compare survival outcomes and the relative cost-effectiveness associated with surgery–radiotherapy and radiotherapy treatment approaches in patients with cervical cancer. A key strength of this study is that all of these analyses were based on real-world survival and economic data. Secondly, subgroup analyses that may have an impact on prognosis may be performed to provide tailored treatment for these cervical cancer patients, improving treatment management in clinical practice, although caution is warranted when interpreting the results of these descriptive analyses given that many included relatively small sample sizes or short follow-up intervals. Thirdly, these results emphasize the fact that population-based economic analyses can be feasibly conducted, providing information that can aid policymakers as they seek to renegotiate treatment pricing, thus leading to lower treatment costs and greater long-term healthcare sustainability. Lastly, the data used herein were based entirely on a Chinese patient population, which is important given that China has one of the highest global cervical cancer rates such that these findings are likely to be broadly applicable.

This study is subject to some limitations. First, retrospective analyses have the potential to lead to selection bias stemming from the non-random nature of treatment assignments and the lack of any effort to control for confounding factors. While a propensity scoring approach was employed to better balance the groups included in this study, the results may nonetheless be subject to these limitations. Second, this study did not place any limitations on tumor size [33], surgical approach, or radiation dose when conducting economic and survival analyses. Future research taking these factors into account has the potential to provide new insight into their predictive relevance in this context. Third, since the number of surgery-related complications observed in this study was relatively small, we did not conduct detailed statistics or present the surgical adverse events. Fourth, the patients in the surgery–radiotherapy group in this study were primarily cervical cancer patients with moderate-/high-risk disease, whereas risk factors other than lymph node status could not be determined for those patients in the radiotherapy group, although the propensity score matching approach was employed to adequately balance the baseline characteristics of individuals in these two groups. Fifth, since no quality-of-life assessment was conducted in the retrospective study, we can only rely on the efficacy values from similar studies. Sixth, the approval date for pembrolizumab in China for its indicated use is December 2024. Prior to this, in the Chinese region, there were relatively few patients with locally advanced cervical cancer who received pembrolizumab treatment, so this study did not include these patients. At present, chemoradiotherapy combined with immunotherapy is the standard treatment for locally advanced cervical cancer, and we are also conducting real-world studies to verify this. Lastly, owing to the nature of the available follow-up data, it was only possible to evaluate the total 8-year costs in both of these treatment groups. Conducting these analyses with a focus on shorter or longer follow-up intervals has the potential to yield different findings, as ICERs can decline with time.

## 5. Conclusions

In summary, the present clinical and economic analyses conducted based on data from a real-world patient population suggest that patients with cervical cancer attain similar survival benefits from surgery–radiotherapy and radiotherapy strategies, with the latter being more cost-effective. On the whole, these findings provide support for oncologists and patient treatment decision-making, maximizing appropriate resource utilization within the healthcare sector while helping guide Medicare-related decisions.

## Figures and Tables

**Table 1:** Baseline demographics and clinical characteristics of the propensity-score-matched population.

	Radiotherapy Group (n = 380)	Surgery–Radiotherapy Group (n = 140)	*p*-Value
**Age, median (range), years**	56 (24–82)	49 (25–71)	0.0899
≥55 years, no. (%)	155 (40.8)	45 (32.1)	
<55 years, no. (%)	225 (59.2)	95 (67.9)	
**Disease stage at initial diagnosis ^a^, no. (%)**			0.465
I–II	188 (49.5)	75 (53.6)	
III–IV	192 (50.5)	65 (46.4)	
**Histologic type, no. (%)**			0.0615
Adenocarcinoma	25 (6.6)	16 (11.4)	
Adenosquamous carcinoma	7 (1.8)	6 (4.3)	
Squamous-cell carcinoma	346 (91.1)	116 (82.9)	
Other ^b^	2 (0.5)	2 (1.4)	
**Differentiation grade ^c^, no. (%)**			0.0873
Well	37 (9.7)	5 (3.6)	
Moderately	228 (60.0)	96 (68.6)	
Poorly	86 (22.6)	31 (22.1)	
Unspecified	29 (7.6)	8 (5.7)	
**HPV status, no. (%)**			0.124
Negative	16 (4.2)	5 (3.6)	
Positive	232 (61.1)	99 (70.7)	
Unspecified	132 (34.7)	36 (25.7)	
**Lymph node metastases, no. (%)**			0.383
Yes	182 (47.9)	62 (44.3)	
Single	40 (10.5)	35 (25.0)	
Multiple	142 (37.4)	27 (19.3)	
No	167 (43.9)	70 (50.0)	
Suspicious ^d^	31 (8.2)	8 (5.7)	
**Combination therapy ^e^, no. (%)**			0.329
Concurrent	352 (92.6)	96 (68.6)	
Neoadjuvant	27 (7.1)	20 (14.3)	
Adjuvant	273 (71.8)	83 (59.3)	
None	24 (6.3)	13 (9.3)	
**RT dose, median (range), Gy**			<0.001
EBRT to pelvis with or without abdomen	45.0 (41.4–59.8)	45.0 (37.8–50.0)	
Brachytherapy (EQD2)	39.7 (29.3–59.5)	18.8 (0–40.0)	
EBRT to lymph node metastases ^f^	60.0 (46.8–65.0)	55.9 (48.4–60.0)	

^a^ Disease stage was determined with the use of International Federation of Gynecology and Obstetrics 2018 (FIGO 2018) [19]. ^b^ Others include neuroendocrine carcinoma, clear cell carcinoma, and gastric adenocarcinoma. ^c^ If the degree of differentiation is mixed, the state with the lower degree of differentiation is selected. ^d^ Radiographic evaluation showed lymph node shadow, but the short diameter did not exceed 1 cm. ^e^ The period of combined therapy includes the concurrent period (which includes chemotherapy [n = 686, 87.9%], targeted therapy [n = 30, 3.8%], and both [n = 25, 3.2%]), the neoadjuvant period (chemotherapy) and the adjuvant period (chemotherapy). ^f^ Contains suspected lymph node metastasis. Abbreviations: HPV, human papilloma virus; RT, radiotherapy; EBRT, external beam radiotherapy; EQD2, equivalent dose in 2 Gy fractions.

**Table 2:** Adverse events of any cause in either group (in a propensity-score-matched treated population).

	Radiotherapy Group (n = 363)		Surgery–Radiotherapy Group (n = 118)	
	Any Grade	Grade ≥ 3	Any Grade	Grade ≥ 3
	*Number of Patients (Percent)*			
**Any event**	361 (99.4)	269 (74.1)	116 (98.3)	59 (50.0)
**Anemia**	337 (92.8)	73 (20.1)	99 (83.9)	13 (11.0)
**Leucopenia**	331 (91.2)	185 (51.0)	106 (89.8)	45 (38.1)
**Hypoalbuminemia**	287 (79.1)	8 (2.2)	83 (70.3)	0 (0)
**Neutropenia**	242 (66.7)	120 (33.1)	87 (73.7)	34 (28.8)
**Thrombocytopenia**	185 (51.0)	34 (9.4)	33 (28.0)	2 (1.7)
**Acute radiation-induced enteritis**	166 (45.7)	42 (11.6)	33 (28.0)	6 (5.1)
**Late radiation-induced enteritis**	112 (30.9)	7 (1.9)	13 (11.0)	2 (1.7)
**Late radiation-induced cystitis**	98 (27.0)	49 (13.5)	8 (6.8)	2 (1.7)
**Elevated AST**	120 (33.1)	5 (1.4)	39 (33.1)	3 (2.5)
**Elevated ALT**	119 (32.8)	7 (1.9)	38 (32.2)	2 (1.7)
**Hypokalemia**	114 (31.4)	13 (3.6)	34 (28.8)	0 (0)
**Nausea/vomiting**	85 (23.4)	0 (0)	29 (24.6)	0 (0)
**Acute radiation-induced cystitis**	77 (21.2)	2 (0.6)	38 (32.2)	1 (0.9)
**Hypocalcemia**	66 (18.2)	0 (0)	11 (9.3)	0 (0)
**Radiodermatitis**	59 (16.3)	0 (0)	9 (7.6)	0 (0)
**Hyponatremia**	57 (15.7)	0 (0)	17 (14.4)	0 (0)
**Elevated serum creatinine**	43 (11.9)	4 (1.1)	1 (0.9)	0 (0)
**Hyperbilirubinemia**	38 (10.5)	3 (0.8)	18 (15.3)	0 (0)
**Proteinuria**	32 (8.8)	0 (0)	9 (7.6)	0 (0)
**Hyperpotassemia**	23 (6.3)	1 (0.3)	0 (0)	0 (0)
**Hypernatremia**	17 (4.7)	0 (0)	0 (0)	0 (0)
**Hypercalcemia**	8 (2.2)	0 (0)	1 (0.9)	0 (0)

Abbreviations: ALT, alanine transaminase; AST, aspartate transaminase.

**Table 3:** Cost-effectiveness results.

Treatment	Cost, Mean 8 y Total, $	Mean LYs	Mean QALYs	Incremental Difference (95% CI)			ICER, $ (95%CI) ^c^	
				Cost, $ ^a^	Lys ^b^	QALYs ^a^	Per Mean LY	Per Mean QALY
**Radiotherapy Group**	26,667	5.531	5.334	2.527 (2.442–2.612)	0.066 (0.051–0.081)	0.062 (0.047–0.077)	Dominated ^d^	40,758 (33,922–51,957)
**Surgery–radiotherapy Group**	29,193	5.465	5.396					

^a^ Surgery–radiotherapy group minus radiotherapy group. ^b^ Radiotherapy group minus surgery–radiotherapy group. ^c^ Surgery–radiotherapy group versus radiotherapy group. ^d^ The surgery–radiotherapy group has lower efficacy and higher cost compared to the radiotherapy group. Abbreviations: LYs, life-years; QALYs, quality-adjusted life-years; ICER, incremental cost-effectiveness ratio.

## Data Availability

All authors had full access to all of the data in this study and take complete responsibility for the integrity of the data and the accuracy of the data analysis. The datasets generated and/or analyzed during the current study are available from the corresponding author upon reasonable request.

## Supplementary Materials

The following supporting information can be downloaded at: https://www.mdpi.com/article/10.3390/cancers18050865/s1, Figure S1: Cohort creation; Figure S2: Kaplan–Meier estimates of overall survival in key subgroups; Figure S3: Kaplan–Meier estimates of progression-free survival in key subgroups; Table S1: List of investigators; Table S2: Baseline demographics and clinical characteristics of overall population; Table S3 [11]: The CHEERS 2022 checklist; Table S4: Summary of response in the efficacy evaluable population; Table S5 [13,14,15,18]: Clinical and health parameters; Table S6: Cost-effectiveness results of subgroup analysis.
