# Supplementary material for: Comprehensive Comparison of Surgery Followed by Radiotherapy and Radical Radiotherapy for Cervical Cancer: A Multicenter Retrospective Propensity-Score-Matched Analysis
Source: Cancers (Basel). 2026 Mar 7;18(5):865. doi: 10.3390/cancers18050865 (PMC12985006; doi:10.3390/cancers18050865)
Supplement: Supplementary file 1 [file cancers-18-00865-s001.zip › cancers-4172694-supplementary.pdf]

Supplementary Appendix

Figure S1: Cohort Creation.
Figure S2. Kaplan-Meier Estimates of Overall Survival in Key Subgroups.
Figure S3. Kaplan-Meier Estimates of Progression-Free Survival in Key Subgroups.
Table S1. List of Investigators
Table S2. Baseline Demographics and Clinical Characteristics of overall population.
Table S3. The CHEERS 2022 checklist.
Table S4. Summary of Response in the Efficacy Evaluable Population.
Table S5. Clinical and Health Parameters.
Table S6. Cost-Effectiveness Results of Subgroup.

Figure S1: Cohort Creation

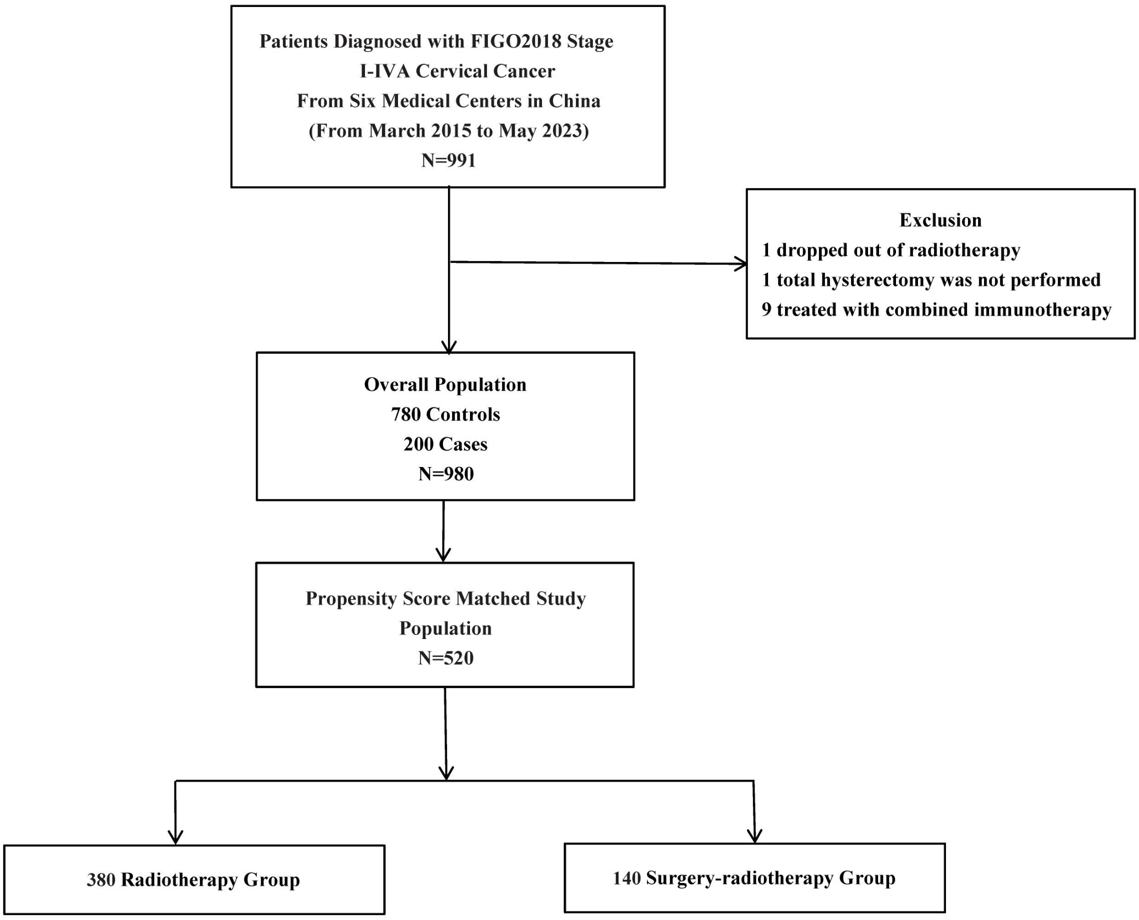

Figure S2. Kaplan-Meier Estimates of Overall Survival in Key Subgroups.

A. < 55 years

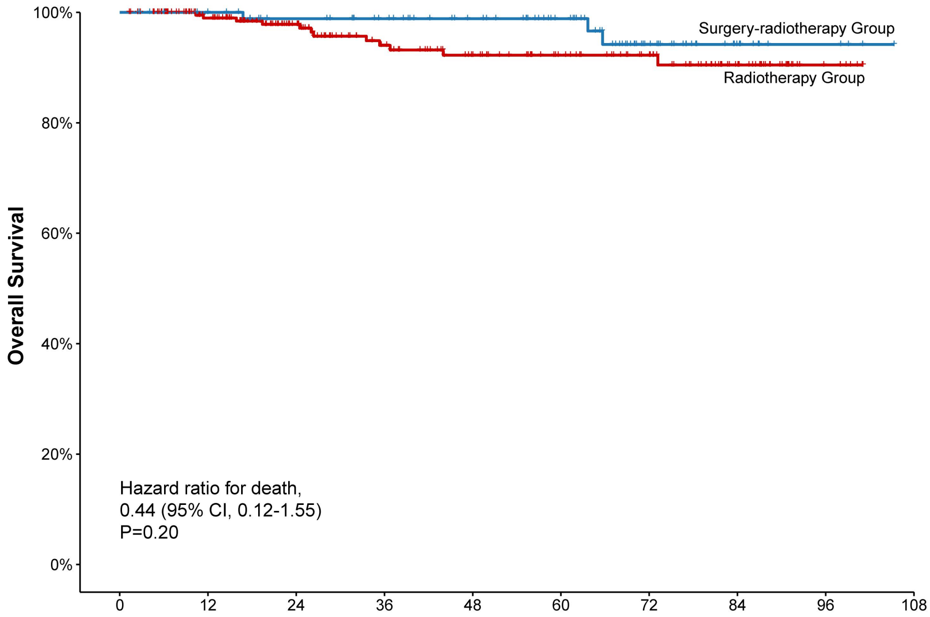

No. at Risk

	0	12	24	36	48	60	72	84	96	108
Radiotherapy Group	225	191	144	112	93	74	59	32	7	0
Surgery-radiotherapy Group	95	90	82	75	65	51	26	10	4	0

B. ≥ 55 years

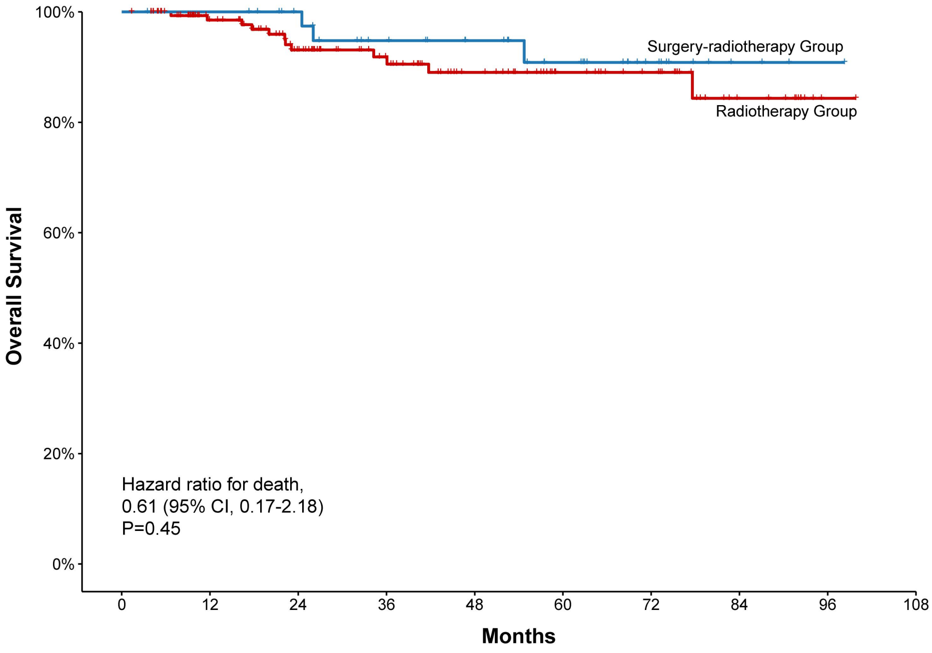

No. at Risk

	0	12	24	36	48	60	72	84	96	108
Radiotherapy Group	155	124	92	71	51	35	28	11	1	0
Surgery-radiotherapy Group	45	44	39	32	27	20	11	3	1	0

C. Squamous-cell carcinoma

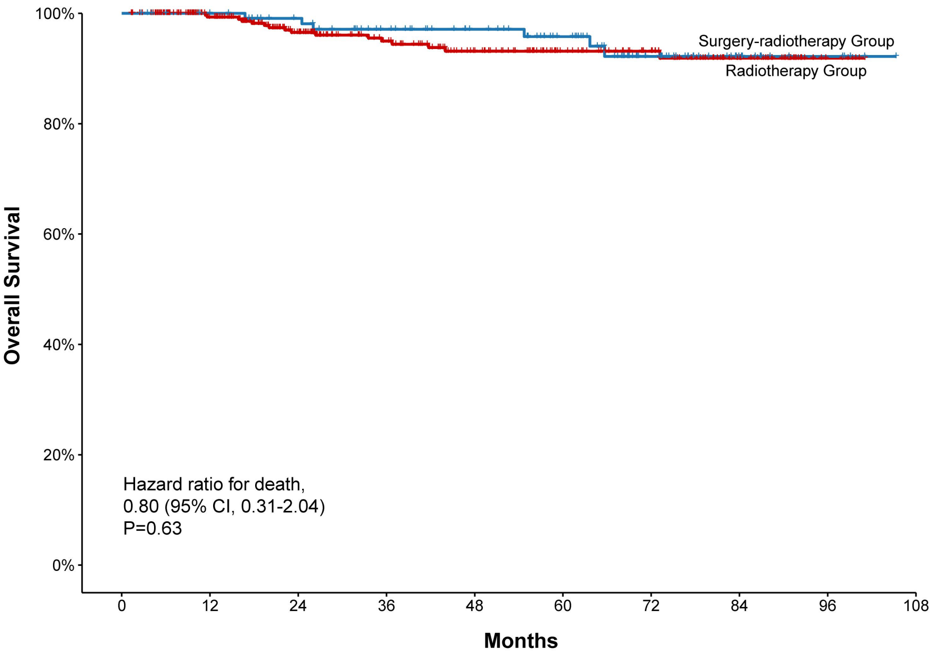

No. at Risk

	0	12	24	36	48	60	72	84	96	108
Radiotherapy Group	346	287	215	170	135	101	81	40	7	0
Surgery-radiotherapy Group	116	110	101	89	77	63	34	12	5	0

D. Adenocarcinoma

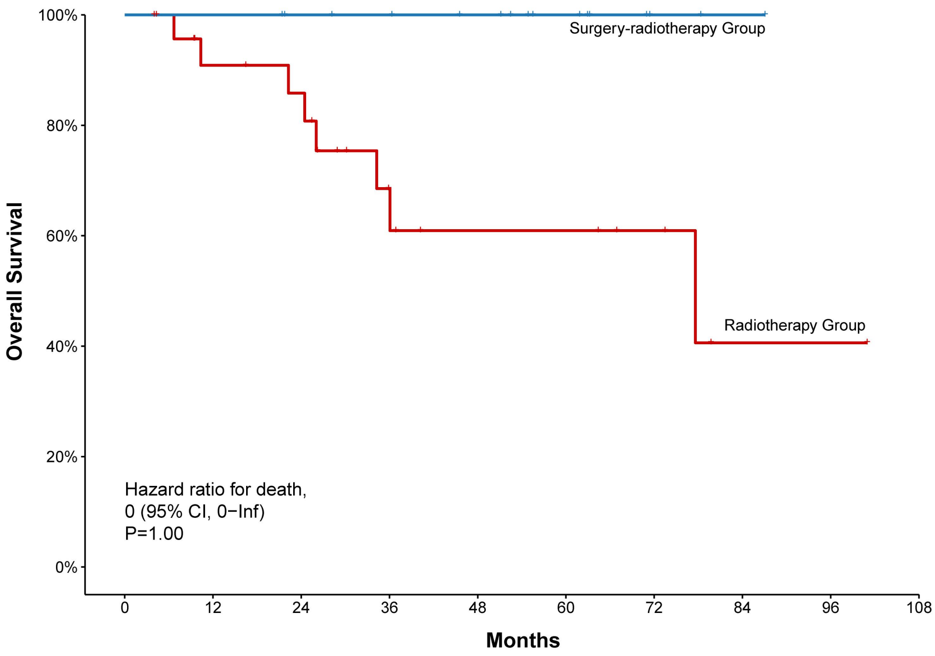

No. at Risk

	0	12	24	36	48	60	72	84	96	108
Radiotherapy Group	25	19	17	9	6	6	4	1	1	0
Surgery-radiotherapy Group	16	16	14	13	11	7	2	1	0	0

E. FIGO 2018 I-IIA2 Stage

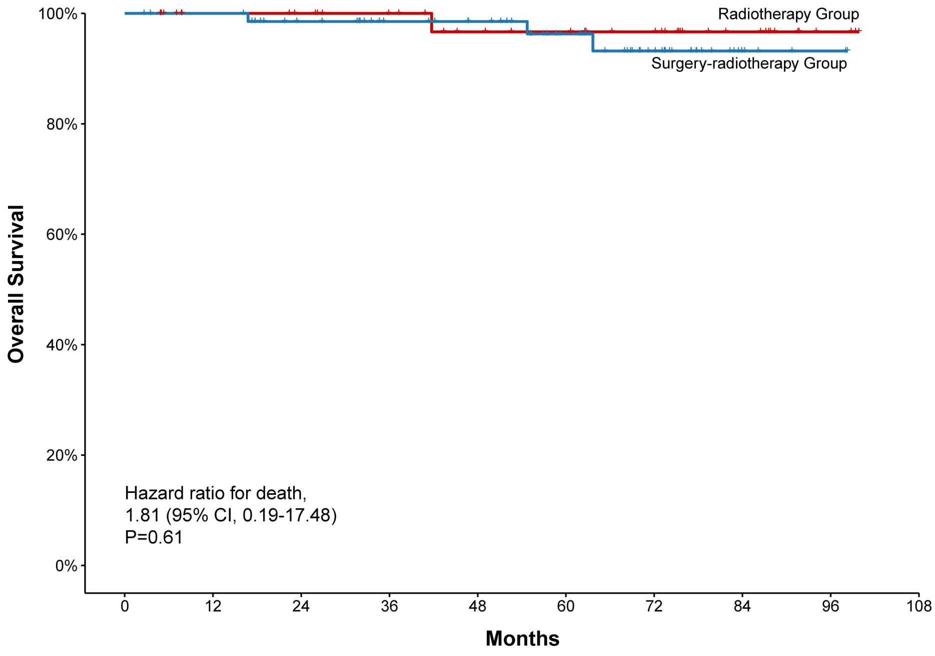

No. at Risk

	0	12	24	36	48	60	72	84	96	108
Radiotherapy Group	44	38	36	32	27	25	21	11	3	0
Surgery-radiotherapy Group	71	69	60	52	48	36	22	5	2	0

F. FIGO 2018 IIB-IVA Stage

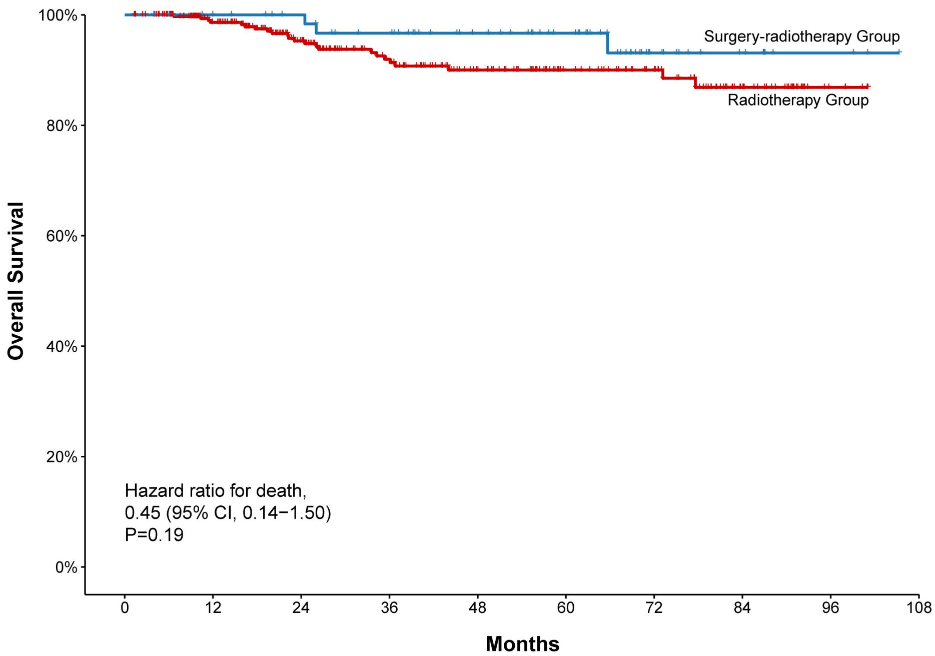

No. at Risk

	0	12	24	36	48	60	72	84	96	108
Radiotherapy Group	336	277	200	151	117	84	66	32	5	0
Surgery-radiotherapy Group	69	65	61	55	44	35	15	8	3	0

G. HPV Positive

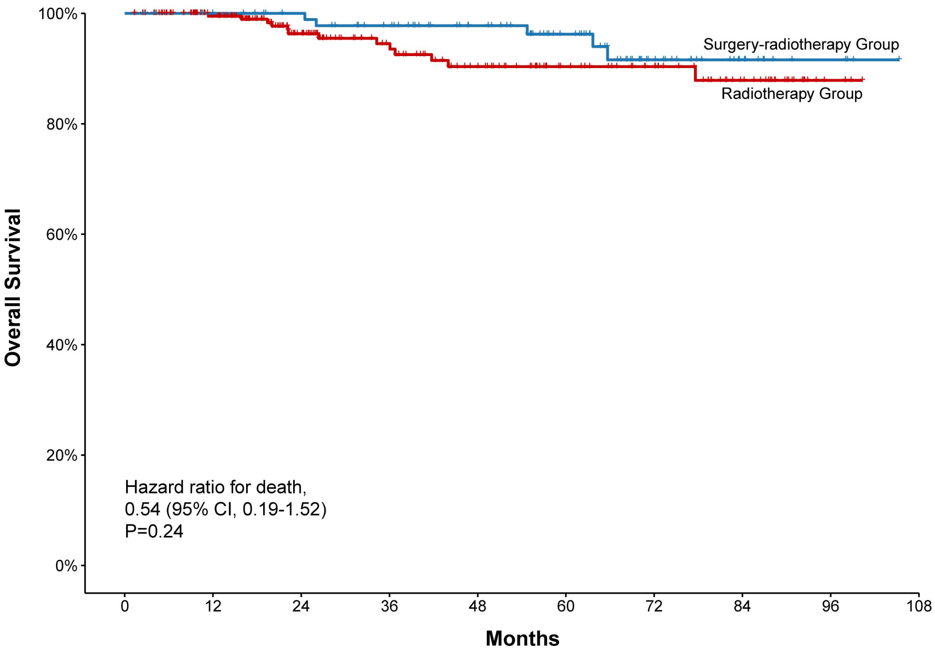

No. at Risk

	0	12	24	36	48	60	72	84	96	108
Radiotherapy Group	232	194	131	96	77	57	44	25	4	0
Surgery-radiotherapy Group	99	95	90	82	69	51	25	11	4	0

H. HPV Negative

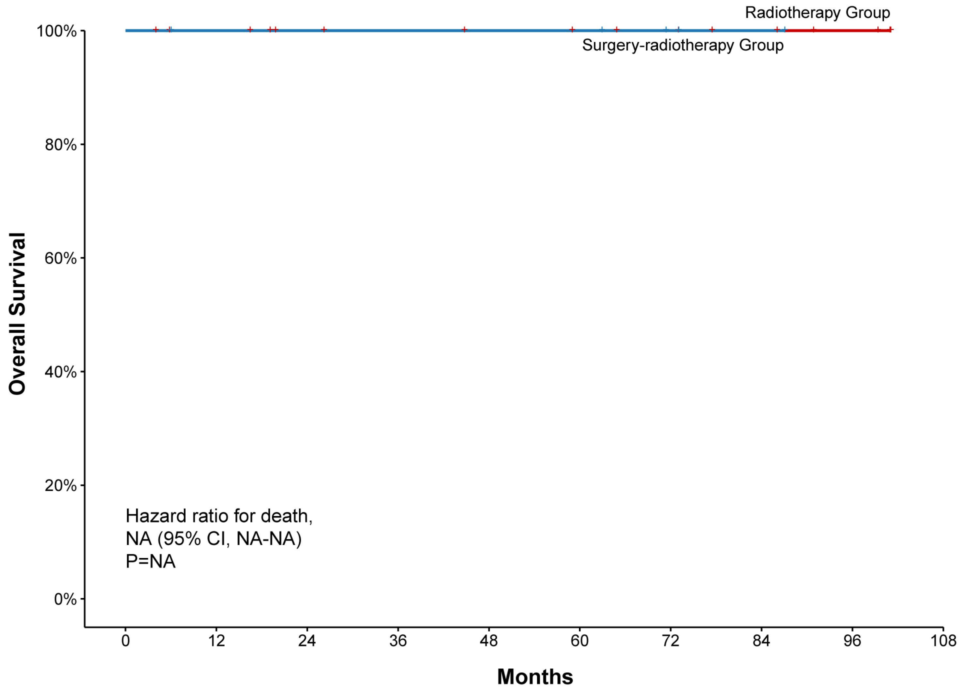

No. at Risk

Radiotherapy Group	16	14	11	10	9	8	7	5	3	0
Surgery-radiotherapy Group	5	4	4	4	4	4	2	1	0	0

I. Lymph Node Metastases Positive

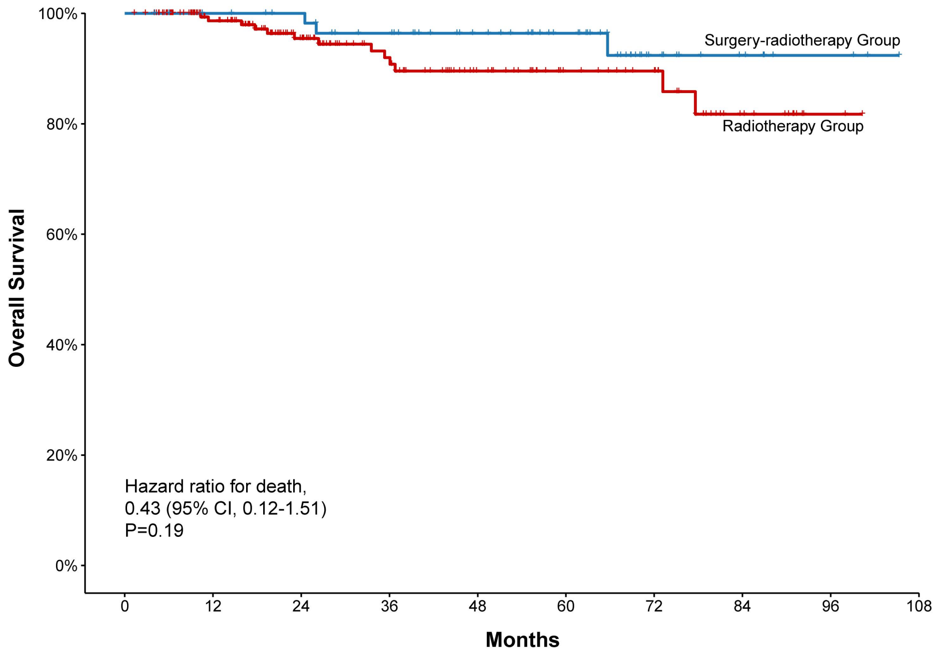

No. at Risk

Radiotherapy Group	182	145	101	76	53	35	29	11	2	0
Surgery-radiotherapy Group	62	59	56	50	40	31	13	7	3	0

J. ⩾4 of Concurrent Therapy Circles

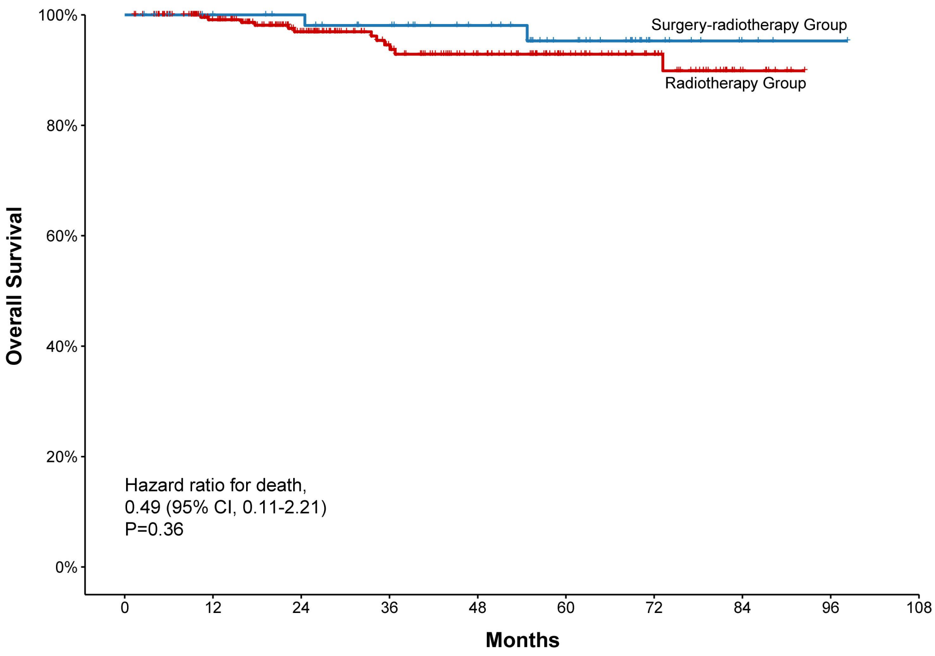

No. at Risk

	0	12	24	36	48	60	72	84	96	108
Radiotherapy Group	266	219	157	114	84	55	37	8	0	0
Surgery-radiotherapy Group	59	54	52	47	39	27	9	3	1	0

K. < 4 of Concurrent Therapy Circles

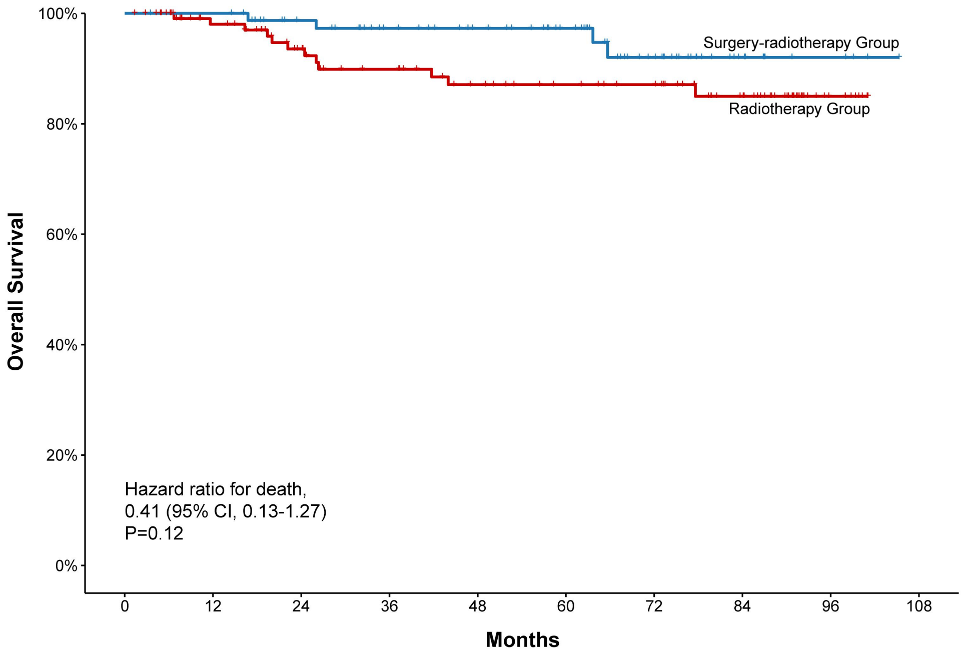

No. at Risk

	0	12	24	36	48	60	72	84	96	108
Radiotherapy Group	114	96	79	69	60	54	50	35	8	0
Surgery-radiotherapy Group	81	80	69	60	53	44	28	10	4	0

L. Adjuvant Chemotherapy

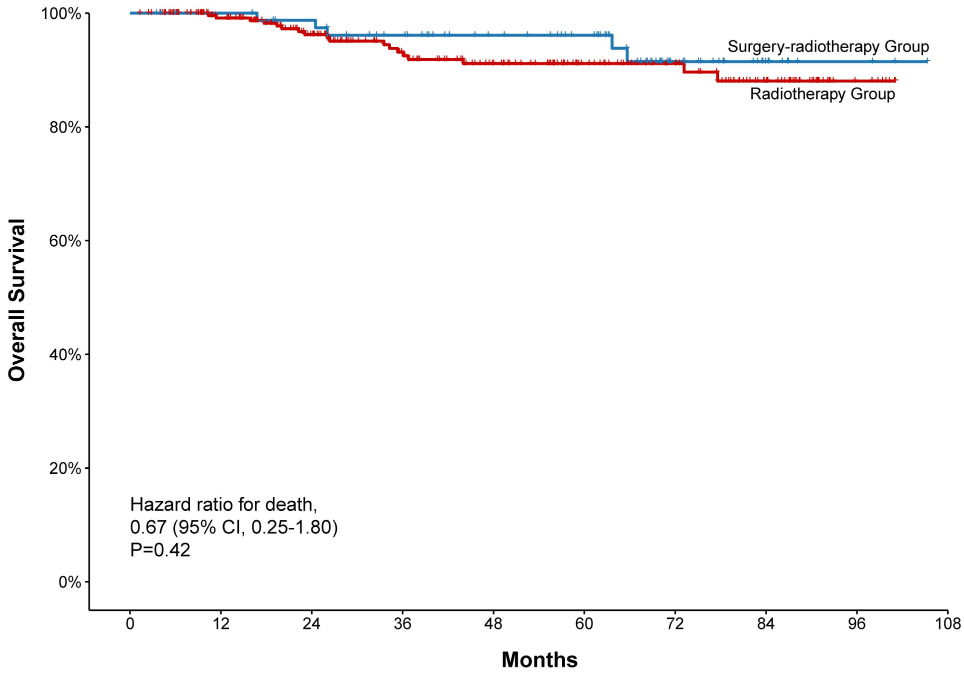

No. at Risk

	0	12	24	36	48	60	72	84	96	108
Radiotherapy Group	273	230	179	143	117	87	67	38	8	0
Surgery-radiotherapy Group	83	80	76	69	59	52	23	9	3	0

M. Neoadjuvant Chemotherapy

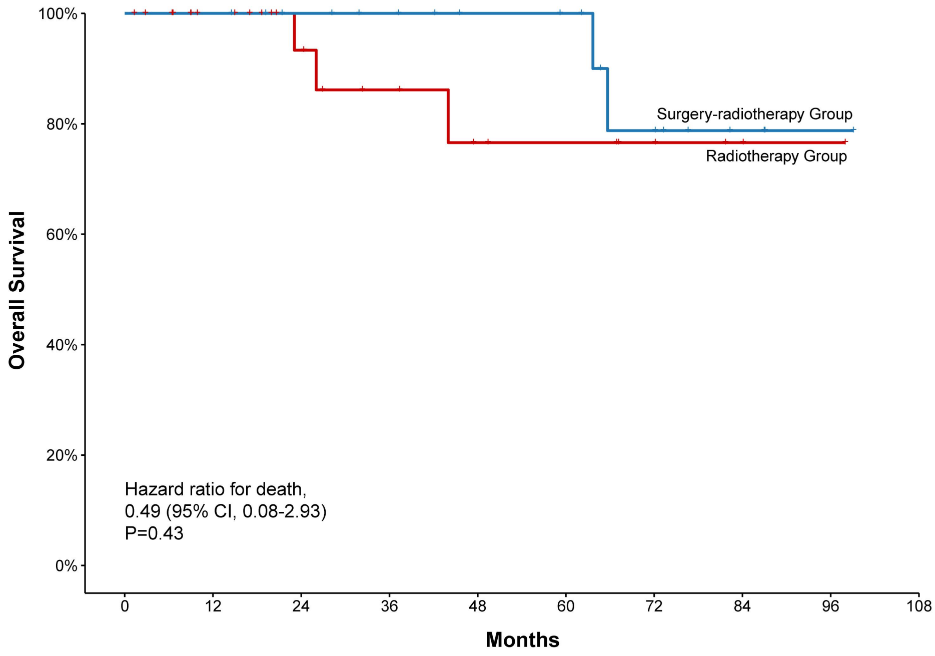

No. at Risk

	0	12	24	36	48	60	72	84	96	108
Radiotherapy Group	27	20	14	10	7	6	4	2	1	0
Surgery-radiotherapy Group	20	20	17	15	12	11	7	3	1	0

Figure S3. Kaplan-Meier Estimates of Progression-Free Survival in Key Subgroups.

A. < 55 years

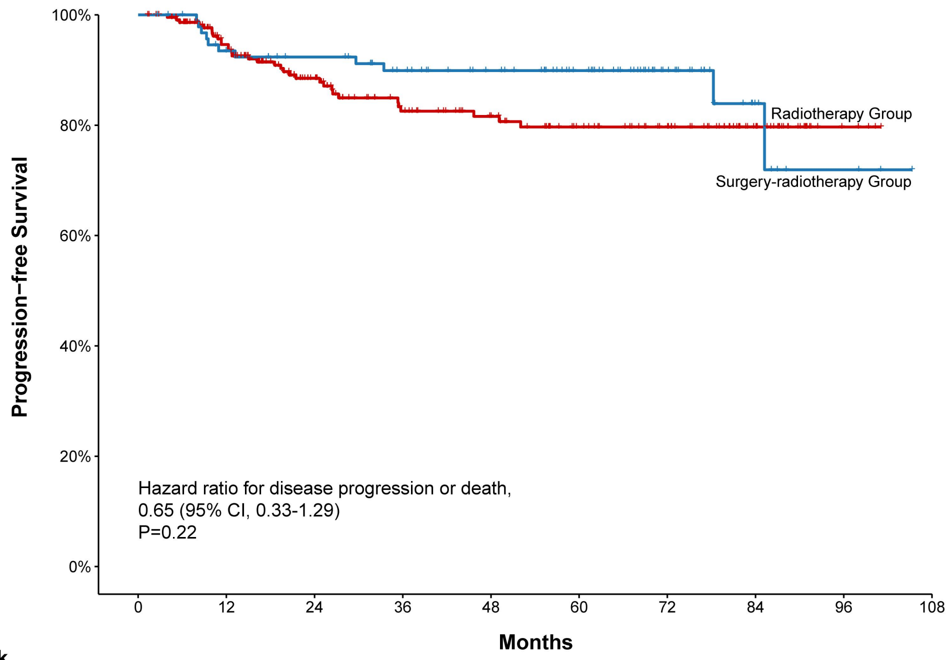

No. at Risk

	0	12	24	36	48	60	72	84	96	108
Radiotherapy Group	225	184	132	102	88	69	54	29	5	0
Surgery-radiotherapy Group	95	84	79	70	61	47	25	8	3	0

B. ≥ 55 yeare

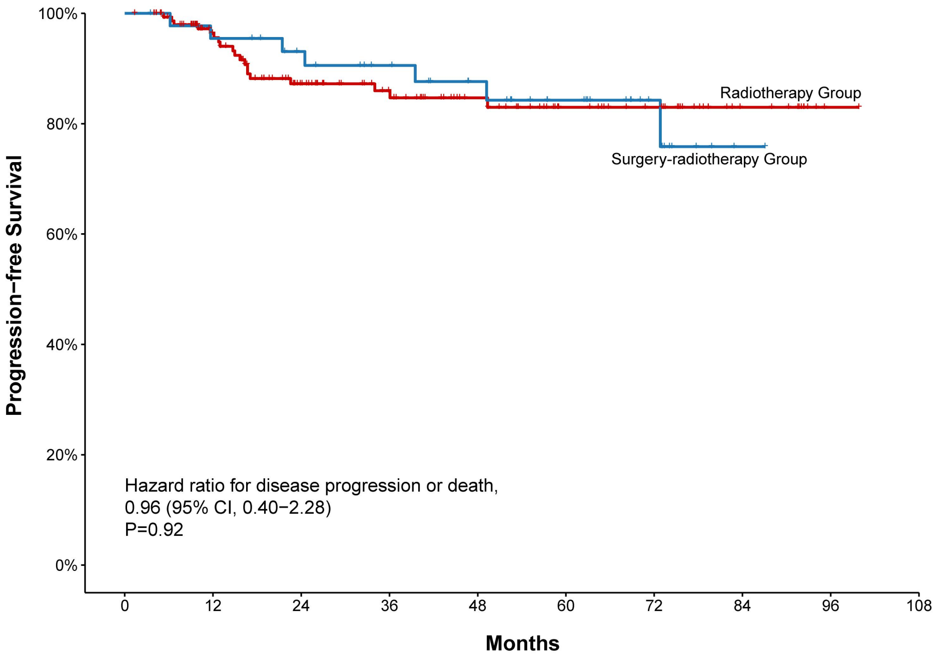

No. at Risk

	0	12	24	36	48	60	72	84	96	108
Radiotherapy Group	155	121	86	67	49	34	27	11	1	0
Surgery-radiotherapy Group	45	42	37	32	26	19	10	1	0	0

C. Squamous-cell carcinoma

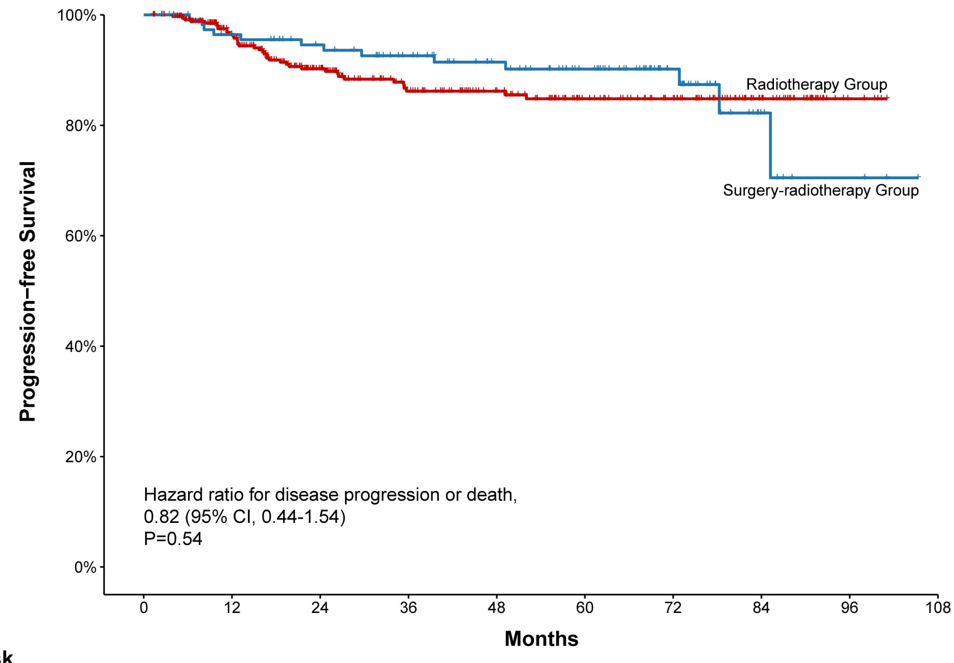

No. at Risk

	0	12	24	36	48	60	72	84	96	108
Radiotherapy Group	346	280	203	158	130	98	78	39	6	0
Surgery-radiotherapy Group	116	106	97	86	73	59	32	8	3	0

D. Adenocarcinoma

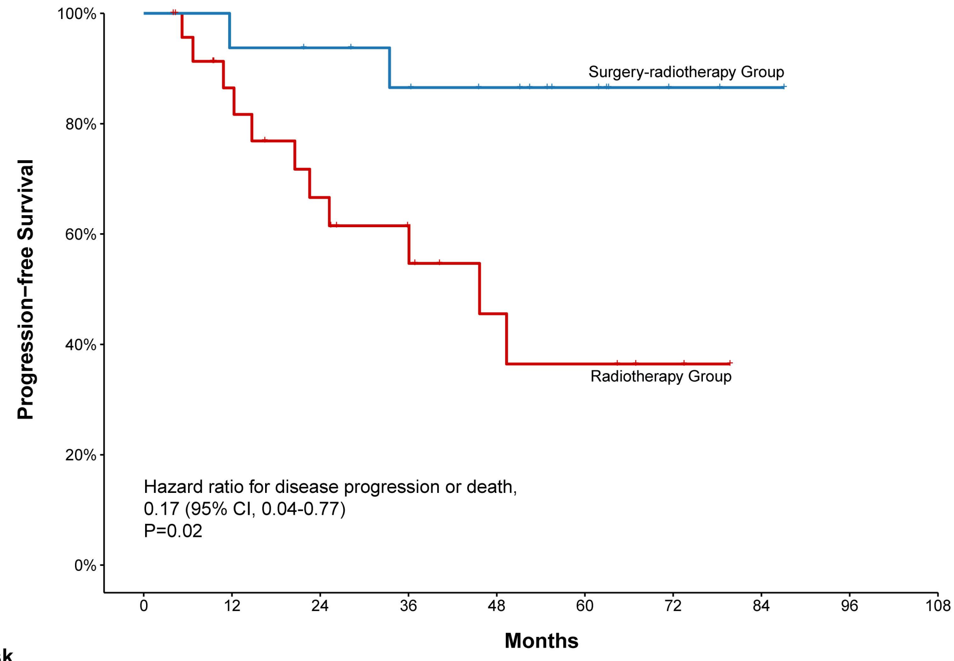

No. at Risk

	0	12	24	36	48	60	72	84	96	108
Radiotherapy Group	25	18	13	9	5	4	2	0	0	0
Surgery-radiotherapy Group	16	15	14	12	10	6	2	1	0	0

E. FIGO 2018 I-IIA2 Stage

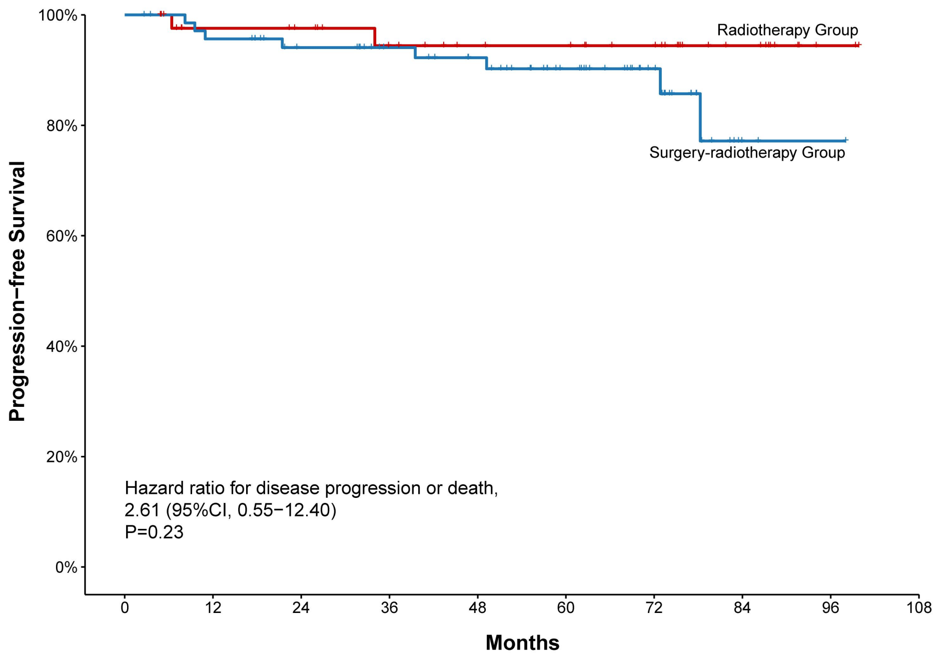

No. at Risk

Radiotherapy Group	44	37	34	29	25	24	20	10	2	0
Surgery-radiotherapy Group	71	66	58	51	46	34	21	2	1	0

F. FIGO 2018 IIB-IVA Stage

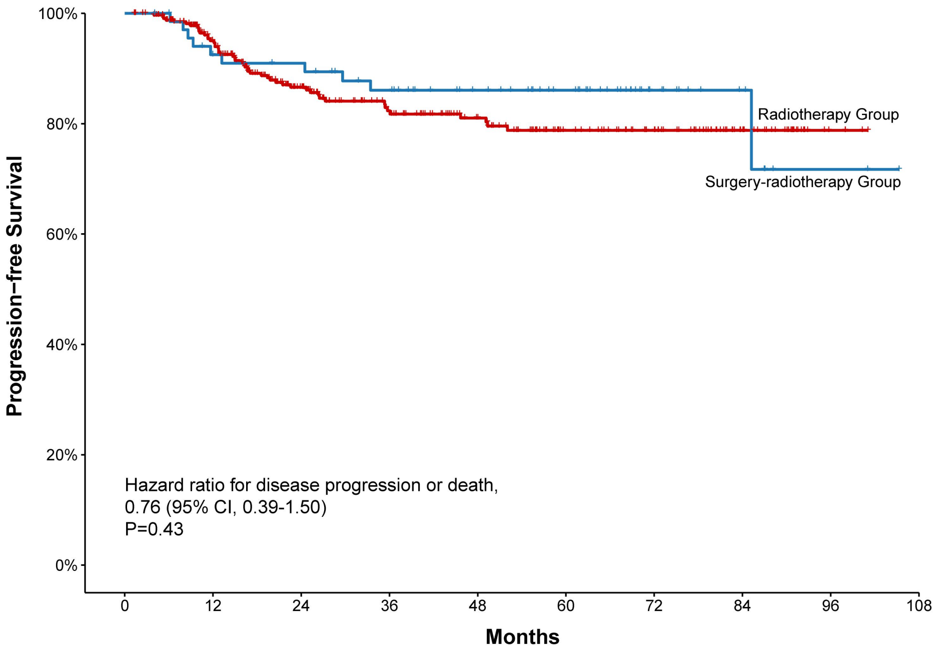

No. at Risk

Radiotherapy Group	336	268	184	140	112	79	61	30	4	0
Surgery-radiotherapy Group	69	60	58	51	41	32	14	7	2	0

G. HPV Positive

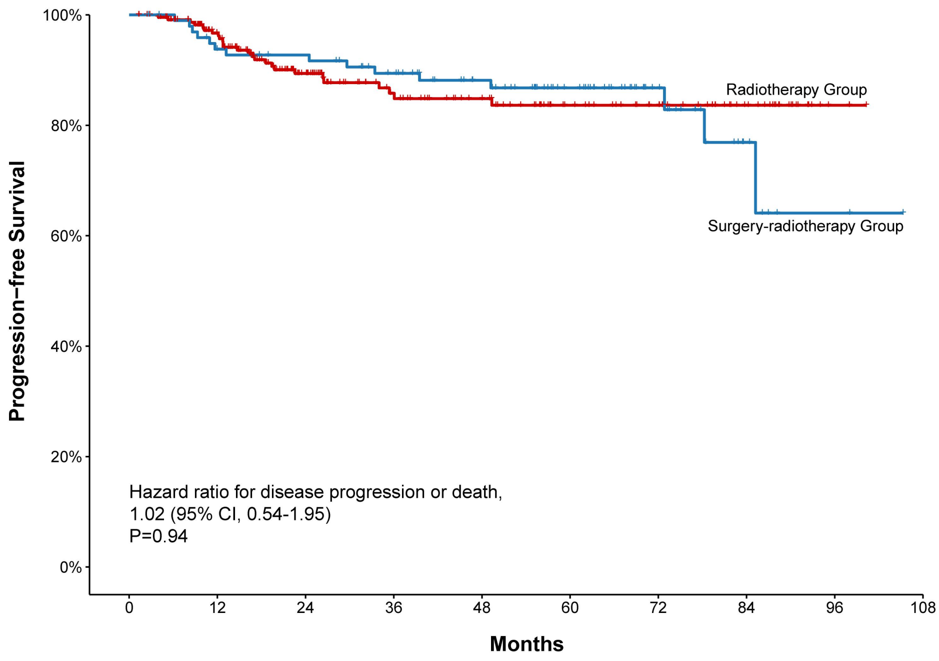

No. at Risk

Radiotherapy Group	232	189	123	88	74	54	41	23	3	0
Surgery-radiotherapy Group	99	89	86	77	64	46	23	7	2	0

H. HPV Negative

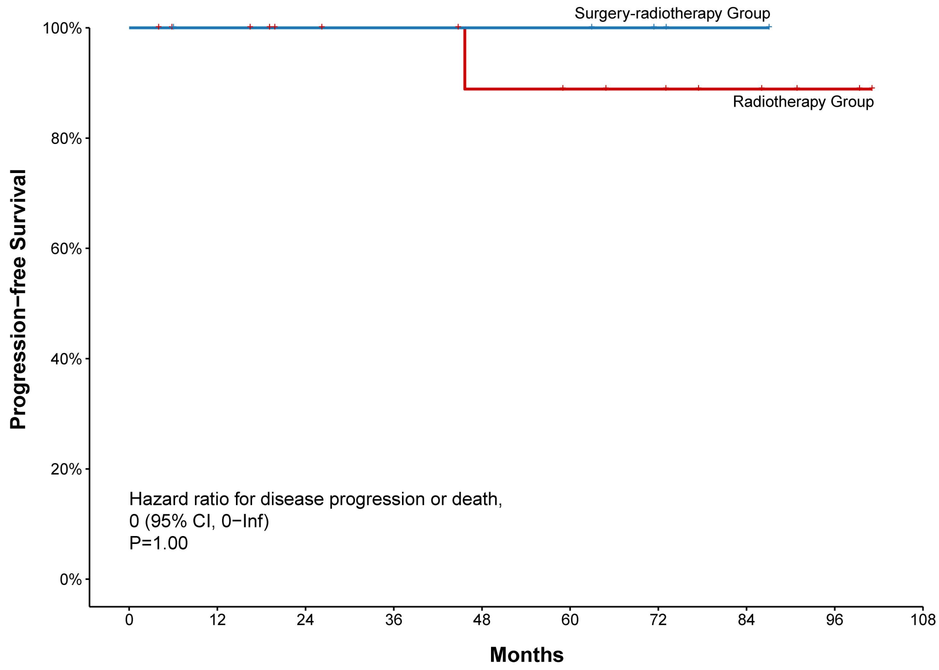

No. at Risk

Radiotherapy Group	16	14	11	10	8	7	6	4	2	0
Surgery-radiotherapy Group	5	4	4	4	4	4	2	1	0	0

I. Lymph Node Metastases Positive

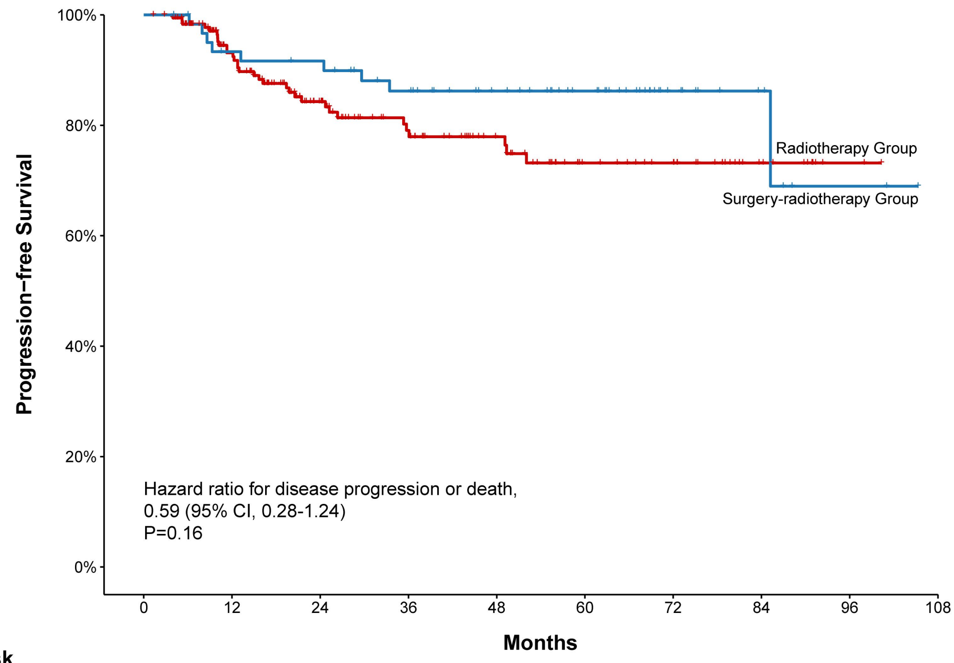

No. at Risk

	0	12	24	36	48	60	72	84	96	108
Radiotherapy Group	182	137	91	69	51	31	25	10	2	0
Surgery-radiotherapy Group	62	55	53	46	37	28	12	6	2	0

J. ⩾4 of Concurrent Therapy Circles

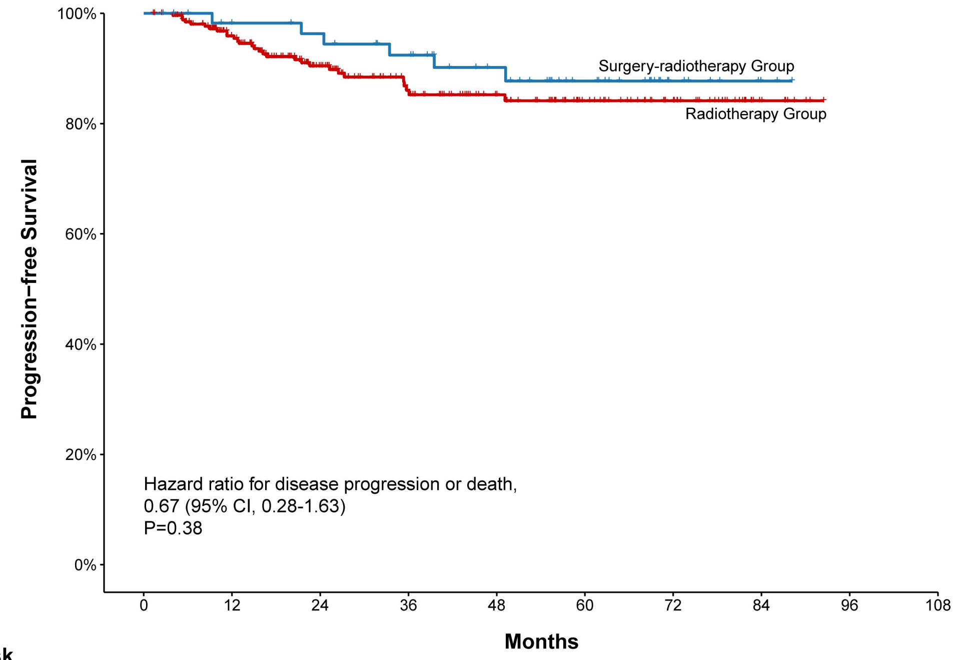

No. at Risk

	0	12	24	36	48	60	72	84	96	108
Radiotherapy Group	266	212	147	106	80	54	36	8	0	0
Surgery-radiotherapy Group	59	53	51	46	37	25	8	2	0	0

K. ＜4 of Concurrent Therapy Circles

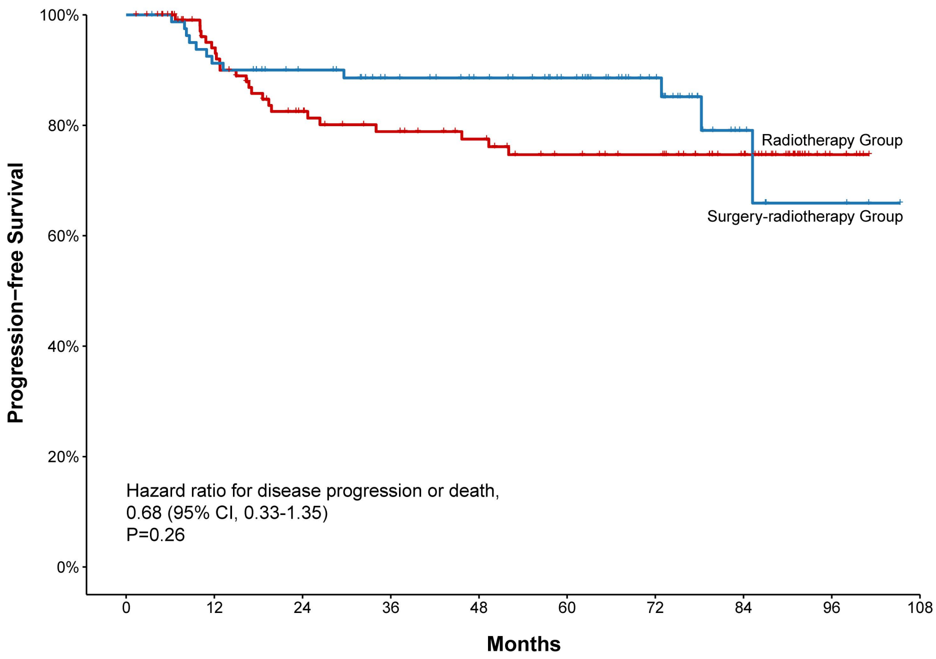

No. at Risk

	0	12	24	36	48	60	72	84	96	108
Radiotherapy Group	114	93	71	63	57	49	45	32	6	0
Surgery-radiotherapy Group	81	73	65	56	50	41	27	7	3	0

L. Adjuvant Chemotherapy

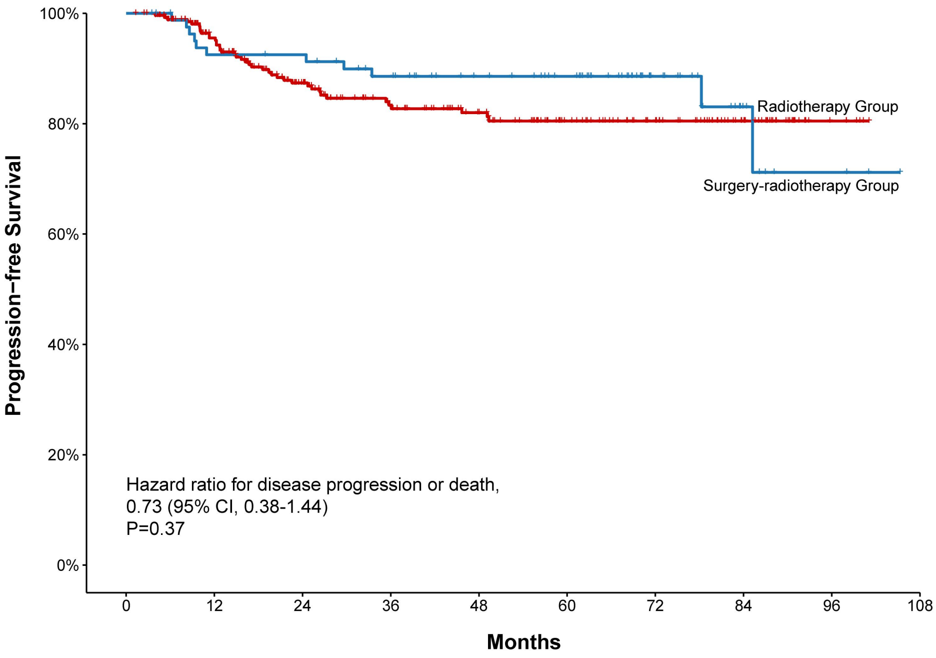

No. at Risk

	0	12	24	36	48	60	72	84	96	108
Radiotherapy Group	273	223	165	132	111	82	62	35	6	0
Surgery-radiotherapy Group	83	74	73	65	56	49	23	8	3	0

M. Neoadjuvant Chemotherapy

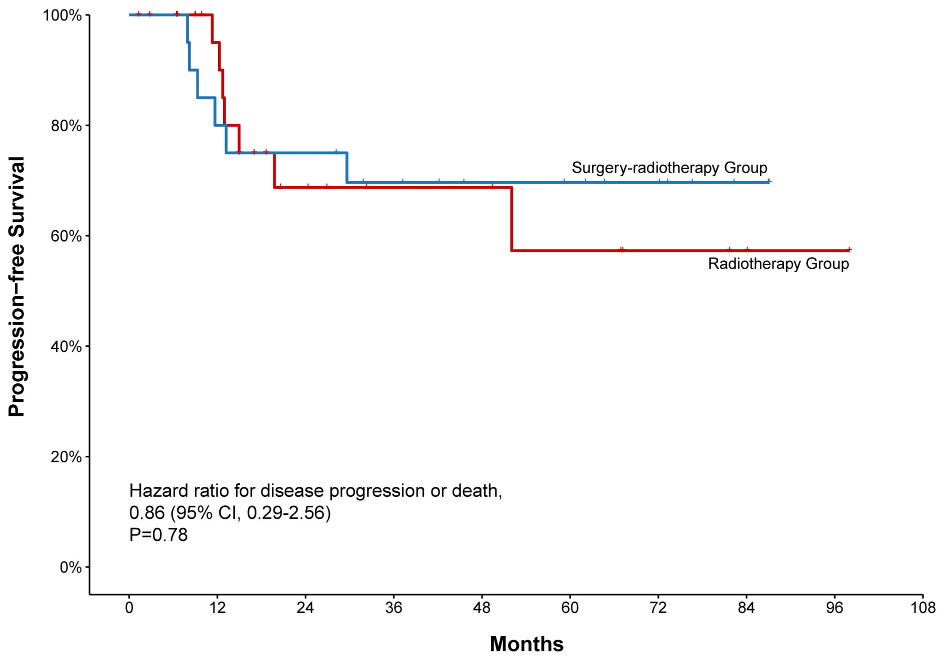

No. at Risk

	0	12	24	36	48	60	72	84	96	108
Radiotherapy Group	27	19	10	7	7	5	3	2	1	0
Surgery-radiotherapy Group	20	16	15	12	9	8	6	2	0	0

Table S1. List of Investigators.

Country/Province/City	Site Name	Principal Investigator
China/Hunan/Changsha	Xiangya Hospital of Central South University	Hong Zhu
	The First Hospital of Hunan University of Chinese Medicine	Weisong Wang
China/Hunan/Loudi	Loudi Central Hospital	Libo Peng
China/Hunan/HuaiHua	Hunan University of Medicine General Hospital	Dongfeng Deng
China/Hebei/Tangshan	Tangshan Cancer Hospital	Qiuping Yang
	Kailuan General Hospital	Yixin Lin

Table S2. Baseline Demographics and Clinical Characteristics of overall population.

	Radiotherapy Group (n=780)	Surgery-radio therapy Group (n=200)	*p*-value
**Age, median (range), years**	56 (24-82)	49 (25-71)	<0.001
≥55 years, no. (%)	416 (53.3)	47 (23.5)	
<55 years, no. (%)	364 (46.7)	153 (76.5)	
**Disease stage at initial diagnosis[a], no. (%)**			0.00942
I-II	425 (54.5)	130 (65.0)	
III-IV	355 (45.5)	70 (35.0)	
**Histologic type, no. (%)**			<0.001
Adenocarcinoma	42 (5.4)	40 (20.0)	
Adenosquamous carcinoma	19 (2.4)	9 (4.5)	
Squamous-cell carcinoma	716 (91.8)	145 (72.5)	
Other[b]	3 (0.4)	6 (3.0)	
**Differentiation grad[c], no. (%)**			0.0224
Well	89 (11.4)	12 (6.0)	
Moderately	464 (59.5)	125 (62.5)	
Poorly	149 (19.1)	50 (25.0)	
Unspecified	78 (10.0)	13 (6.5)	
**HPV status, no. (%)**			0.0877
Negative	30 (3.8)	13 (6.5)	

Positive	507 (65.0)	137 (68.5)	
Unspecified	243 (31.2)	50 (25.0)	
**Lymph node metastases, no. (%)**			<0.001
Yes	233 (29.9)	67 (33.5)	
Single	56 (7.2)	35 (17.5)	
Multiple	177 (22.7)	32 (16.0)	
No	251(32.2)	125(62.5)	
Suspicious[d]	296 (37.9)	8 (4.0)	
**Combination therapy, no. (%)**			<0.001
Concurrent	741 (95.0)	110 (55.0)	
Chemotherapy	686 (87.9)	109 (54.5)	
Targeted therapy	30 (3.8)	1 (0.5)	
Both	25 (3.2)	0 (0)	
Neoadjuvant	41 (5.3)	21 (10.5)	
Adjuvant	554 (71.0)	97 (48.5)	
None	32 (4.1)	51 (25.5)	
**RT dose, median (range), Gy**			<0.001
EBRT to pelvis with or without abdomen	45.0 (39.6-59.8)	45.0 (37.8-50.0)	
Brachytherapy to primary lesion (EQD2)	39.7 (29.3-59.5)	18.8 (0-40.0)	
EBRT to lymph node metastases[e]	59.8 (46.8-67.5)	55.9 (48.4-60.0)	

[a] Disease stage was determined with the use of International Federation of Gynecology and Obstetrics 2018 (FIGO 2018).

[b] Others include neuroendocrine carcinoma, clear cell carcinoma, and gastric adenocarcinoma.

[c] If the degree of differentiation is mixed, the state with the lower degree of differentiation is selected.

[d] Radiographic evaluation showed lymph node shadow but the short diameter did not exceed 1cm.

[e] Contains suspected lymph node metastasis.

Abbreviation: HPV, human papilloma virus; RT, radiotherapy; EBRT, external beam radiotherapy; EQD2, equivalent Dose in 2-Gy fractions.

Table S3. The CHEERS 2022 checklist.

Section/item	Item No	Recommendation	Reported?
**Title and abstract**			
Title	1	Identify the study as an economic evaluation and specify the interventions being compared.	Yes
Abstract	2	Provide a structured summary that highlights context, key methods, results, and alternative analyses.	Yes
**Introduction**			
Background and objectives	3	Give the context for the study, the study question, and its practical relevance for decision making in policy or practice.	Yes
**Methods**			
Health economic analysis plan	4	Indicate whether a health economic analysis plan was developed and where available.	Yes
Study population	5	Describe characteristics of the study population (such as age range, demographics, socioeconomic, or clinical characteristics).	Yes
Setting and location	6	Provide relevant contextual information that may influence findings.	Yes
Comparators	7	Describe the interventions or strategies being compared and why chosen.	Yes
Perspective	8	State the perspective(s) adopted by the study and why chosen.	Yes
Time horizon	9	State the time horizon for the study and why appropriate.	Yes
Discount rate	10	Report the discount rate(s) and reason chosen.	Yes
Selection of outcomes	11	Describe what outcomes were used as the measure(s) of benefit(s) and harm(s)	Yes
Measurement of outcomes	12	Describe how outcomes used to capture benefit(s) and harm(s) were measured.	Yes
Valuation of outcomes	13	Describe the population and methods used to measure and value outcomes.	Yes

Measurement and valuation of resources and costs	14	Describe how costs were valued.	Yes
Currency, price date, and conversion	15	Report the dates of the estimated resource quantities and unit costs, plus the currency and year of conversion.	Yes
Rationale and description of model	16	If modelling is used, describe in detail and why used. Report if the model is publicly available and where it can be accessed.	Yes
Analytics and assumptions	17	Describe any methods for analysing or statistically transforming data, any extrapolation methods, and approaches for validating any model used.	Yes
Characterizing heterogeneity	18	Describe any methods used for estimating how the results of the study vary for subgroups.	Yes
Characterizing distributional effects	19	Describe how impacts are distributed across different individuals or adjustments made to reflect priority populations.	Yes
Characterizing uncertainty	20	Describe methods to characterise any sources of uncertainty in the analysis.	Yes
Approach to engagement with patients and others affected by the study	21	Describe any approaches to engage patients or service recipients, the general public, communities, or stakeholders (such as clinicians or payers) in the design of the study.	Not applicable
**Results**			
Study parameters	22	Report all analytic inputs (such as values, ranges, references) including uncertainty or distributional assumptions.	Yes
Summary of main results	23	Report the mean values for the main categories of costs and outcomes of interest and summarise them in the most appropriate overall measure.	Yes
Effect of uncertainty	24	Describe how uncertainty about analytic judgments, inputs, or projections affect findings. Report the effect of choice of discount rate and time horizon, if applicable.	Yes

Effect of engagement with patients and others affected by the study	25	Report on any difference patient/service recipient, general public, community, or stakeholder involvement made to the approach or findings of the study.	Not applicable
**Discussion**			
Study findings, limitations, generalizability, and current knowledge	26	Report key findings, limitations, ethical or equity considerations not captured, and how these could affect patients, policy, or practice.	Yes
**Other**			
Source of funding	27	Describe how the study was funded and any role of the funder in the identification, design, conduct, and reporting of the analysis	Yes
Conflicts of interest	28	Report authors conflicts of interest according to journal or International Committee of Medical Journal Editors requirements.	Yes

Reference:

1. Husereau D, Drummond M, Augustovski F, de Bekker-Grob E, Briggs AH, Carswell C, et al. Consolidated Health Economic Evaluation Reporting Standards 2022 (CHEERS 2022) Statement: Updated Reporting Guidance for Health Economic Evaluations. Value Health. 2022;25(1):3-9.

Table S4. Summary of Response in the Efficacy Evaluable Population.

	Radiotherapy Group (n=380)	Surgery-radiotherapy Group (n=140)
**ORR, no. (%) [95% CI]**	331 (87.1) [83.7-90.5]	123 (87.9) [82.4-93.3]
**DCR, no. (%) [95% CI]**	335 (88.2) [84.9-91.5]	123 (87.9) [82.4-93.3]
**Best overall response, no. (%)**		
CR	313 (82.4)	123 (87.9)
PR	20 (5.3)	0 (0)
SD	2 (0.5)	0 (0)
PD	45 (11.8)	18 (12.9)

Abbreviation: ORR, objective response rate; CI, confidence interval; DCR, disease control rate; CR, complete response; PR, partial response; SD, stable disease; PD, progressive disease.

Table S5. Clinical and Health Parameters.

Variable	Baseline value (Range)[a]	Distribution
**Clinical Parameters**		
**Hazard ratio (SR vs. R)**		
OS	0.490 (0.200-1.210)	NA
PFS	0.750 (0.440-1.280)	NA
**Risk for main AEs in R group**		
Anemia	0.201 (0.161-0.241)	Beta
Acute radiation-induced enteritis	0.116 (0.093-0.139)	Beta
Late radiation-induced cystitis	0.135 (0.108-0.162)	Beta
Leucopenia	0.510 (0.408-0.612)	Beta
Neutropenia	0.331 (0.265-0.397)	Beta
Thrombocytopenia	0.094 (0.075-0.113)	Beta
**Risk for main AEs in SR group**		
Anemia	0.110 (0.088-0.132)	Beta
Leucopenia	0.381 (0.305-0.457)	Beta
Neutropenia	0.288 (0.230-0.346)	Beta
**Body weight, Kg**	65 (52-78)[1]	Uniform
**Body surface area, Meters^2**	1.720 (1.376-2.064)[1]	Uniform
**Health Parameters**		
**Utility and disutility**		

Utility of PFS	0.820 (0.656-0.984)[2, 3]	Beta
Utility of PD	0.650 (0.520-0.780)[2, 3]	Beta
**Discount rate**	0.05 (0-0.08)[1]	Uniform
**Cost Parameters**		
Radiotherapy (include brachytherapy)	9,724 (7,779-11,669)	Gamma
Postoperative radiotherapy (include brachytherapy)	8,335 (6,668-10,002)	Gamma
Total abdominal hysterectomy	3,316 (2,653-3,979)	Gamma
Total laparoscopic hysterectomy	2,651 (2,121-3,181)	Gamma
Concurrent chemotherapy	70 (56-84)	Gamma
Concurrent nimotuzumab	3,987 (3,190-4,784)	Gamma
Neoadjuvant or adjuvant	834 (667-1,001)	Gamma
AEs of radiotherapy group	85 (68-102)[4]	Gamma
AEs of surgery-radiotherapy group	29 (23-35)[4]	Gamma
Laboratory tests per cycle	R, 208 (166-250)<br>SR, 278 (222-334)	Gamma
Imaging per cycle	347 (277-416)	Gamma
Administration per cycle	195 (156-234)	Gamma
Hospital fees per patient	126 (101-151)	Gamma

[a] The main clinical and cost parameters were obtained from Xiangya Hospital of Central South University and other included hospitals.

Abbreviation: SR, surgery-radiotherapy group; R, radiotherapy group; OS, overall survival; NA, not applicable; PFS, progression-free survival; AEs, adverse events; PD, progressive disease.

References

1. Zhu Y, Liu K, Zhu H, Wu H. Immune checkpoint inhibitors plus chemotherapy for HER2-negative advanced gastric/gastroesophageal junction cancer: a cost-effectiveness analysis. Therap Adv Gastroenterol. 2023 Nov 2;16:17562848231207200.

2. Kim H, Rajagopalan MS, Beriwal S, Huq MS, Smith KJ. Cost-effectiveness analysis of 3D image-guided brachytherapy compared with 2D brachytherapy in the treatment of locally advanced cervical cancer. Brachytherapy. 2015;14(1):29-36.

3. Jewell EL, Smrtka M, Broadwater G, Valea F, Davis DM, Nolte KC, et al. Utility scores and treatment preferences for clinical early-stage cervical cancer. Value Health. 2011;14(4):582-6.

4. Zhu Y, Liu K, Qin Q, Zhu H. Serplulimab plus chemotherapy as first-line treatment for extensive-stage small-cell lung cancer: A cost-effectiveness analysis. Front Immunol. 2022;13:1044678.

Table S6. Cost-Effectiveness Results of Subgroup.

Subgroup	OS HR (95% CI)[a]	PFS HR (95% CI)[a]	ICER, $/QALY	Cost-effectiveness probability at WTP of 35,841/QALY, %	
				Radiotherapy Group	Surgery-radiotherapy Group
**Age, years**					
≥55 years	0.61 (0.17-2.18)	0.96 (0.40-2.28)	97,139	74.04	25.96
<55 years	0.44 (0.12-1.55)	0.65 (0.33-1.29)	41,076	51.43	48.57
**Disease stage**					
I-IIA2	1.81 (0.19-17.48)	2.61 (0.55-12.40)	3,960	76.06	24.85
IIB-IVA	0.45 (0.14-1.50)	0.76 (0.39-1.50)	49,312	56.68	43.32
**Histologic type**					
Adenocarcinoma[a]	0.49 (0.20-1.21)	0.17 (0.04-0.77)	29,551	44.45	55.55
Adenosquamous carcinoma[a]	0.49 (0.20-1.21)	0.34 (0.04-3.29)	32,446	49.48	50.52
Squamous-cell carcinoma	0.80 (0.31-2.04)	0.82 (0.44-1.54)	78,523	65.19	34.81

**HPV status**					
Positive	0.54 (0.19-1.52)	1.02 (0.54-1.95)	112,891	74.63	25.37
Negative[a]	0.49 (0.20-1.21)	0.75 (0.44-1.28)	48,568	55.24	44.76
**Lymph node metastases**					
Yes	0.43 (0.12-1.51)	0.59 (0.28-1.24)	38,352	51.33	48.67
No	0.28 (0.04-2.30)	0.91 (0.35-2.36)	53,355	60.56	39.44
Suspicious	1.02 (0.18-5.75)	1.55 (0.38-6.25)	Dominated[c]	100	0
**Number of lymph node metastases**					
Single	0.44 (0.04-4.86)	1.14 (0.30-4.34)	217,184	82.69	17.31
Multiple	0.59 (0.13-2.66)	0.50 (0.18-1.43)	38,372	51.98	48.02
**Concurrent therapy circles**					
≥4	0.49 (0.11-2.21)	0.67 (0.28-1.63)	43,438	52.52	47.48
<4	0.41 (0.13-1.27)	0.68 (0.33-1.35)	41,723	52,28	4772

**Neoadjuvant chemotherapy**					
Yes	0.49 (0.08-2.93)	0.86 (0.29-2.57)	59,641	61.03	38.97
No	0.42 (0.15-1.24)	0.62 (0.33-1.18)	39,297	50.60	49.40
**Adjuvant chemotherapy**					
Yes	0.67 (0.25-1.80)	0.75 (0.38-1.44)	56,794	58.89	41.11
No	0.21 (0.03-1.73)	0.80 (0.31-2.05)	42,265	52.01	47.99
**Differentiation grad**					
Well[a]	0.49 (0.20-1.21)	0.75 (0.44-1.28)	48,568	55.24	44.76
Moderately	0.49 (0.16-1.48)	0.57 (0.27-1.21)	38,878	51.99	48.01
Poorly	0.57 (0.12-2.74)	1.12 (0.45-2.74)	370,772	86.83	13.17

[a] HR unavailable in partial subgroup, assumed to be consistent with the HR for overall patients.
[c] Surgery-radiotherapy group has lower efficacy and higher cost compared to radiotherapy group.

Abbreviation: OS, overall survival; HR, hazard ratio; CI, confidence interval; PFS, progression-free survival; ICER, incremental cost-effectiveness ratio; QALY, quality-adjusted life-year; HPV, human papillomavirus.
